# Diversity of sulfur cycling halophiles within the Salton Sea, California’s largest lake

**DOI:** 10.1186/s12866-025-03839-2

**Published:** 2025-03-06

**Authors:** Linton Freund, Caroline Hung, Talyssa M. Topacio, Charles Diamond, Alyson Fresquez, Timothy W. Lyons, Emma L. Aronson

**Affiliations:** 1 Genetics, Genomics, and Bioinformatics Program, University of California, Riverside, 900 University Ave, Riverside, CA 92521 USA; 2 Department of Earth and Planetary Sciences, University of California, Riverside, 900 University Ave, Riverside, CA 92521 USA; 3 Department of Microbiology and Plant Pathology, University of California, Riverside, 900 University Ave, Riverside, CA 92521 USA

**Keywords:** Metagenomics, Amplicon sequencing, Sulfur cycling, Sulfur oxidation

## Abstract

**Background:**

Microorganisms are the biotic foundation for nutrient cycling across ecosystems, and their assembly is often based on the nutrient availability of their environment. Though previous research has explored the seasonal lake turnover and geochemical cycling within the Salton Sea, California’s largest lake, the microbial community of this declining ecosystem has been largely overlooked. We collected seawater from a single location within the Salton Sea at 0 m, 3 m, 4 m, 5 m, 7 m, 9 m, 10 m, and 10.5 m depths in August 2021, December 2021, and April 2022.

**Results:**

We observed that the water column microbiome significantly varied by season (*R*^2^ = 0.59, *P* = 0.003). Temperature (*R*^2^ = 0.27, *P* = 0.004), dissolved organic matter (*R*^2^ = 0.13, *P* = 0.004), and dissolved oxygen (*R*^2^ = 0.089, *P* = 0.004) were significant drivers of seasonal changes in microbial composition. In addition, several halophilic mixotrophs and other extremotolerant bacteria were consistently identified in samples across depths and time points, though their relative abundances fluctuated by season. We found that while sulfur cycling genes were present in all metagenomes, their relative coverages fluctuated by pathway and season throughout the water column. Sulfur oxidation and incomplete sulfur oxidation pathways were conserved in the microbiome across seasons.

**Conclusions:**

Our work demonstrates that the microbiome within the Salton Seawater has the capacity to metabolize sulfur species and utilize multiple trophic strategies, such as alternating between chemorganotrophy and chemolithoautrophy, to survive this harsh, fluctuating environment. Together, these results suggest that the Salton Sea microbiome is integral in the geochemical cycling of this ever-changing ecosystem and thus contributes to the seasonal dynamics of the Salton Sea. Further work is required to understand how these environmental bacteria are implicated relationship between the Salton Sea’s sulfur cycle, dust proliferation, and respiratory distress experienced by the local population.

**Supplementary Information:**

The online version contains supplementary material available at 10.1186/s12866-025-03839-2.

## Introduction

The Salton Sea is a terminal, hypersaline lake located in Southern California that receives agricultural runoff as its main source of inflow [1]. This lake formed from the flooding of an irrigation canal in 1905, which diverted Colorado River water into the basin [1, 2]. Though the Salton Sea began as a freshwater lake, it quickly became saline as its waters dissolved the halite crust left behind by ancient Lake Cahuilla [1]. Currently, the Salton Sea is a hypersaline (55 -70 ppt; 3), polluted lake that is rapidly shrinking. Since the Quantification Settlement Agreement in 2003, Colorado River water intended for farms in Imperial County was diverted to support growing populations in Southern California, reducing freshwater input into the Sea [4]. Additionally, the agricultural runoff coming from the New, Alamo, and Whitewater Rivers introduces high concentrations of nitrogen, phosphorous, and sulfur into the Salton Sea, along with a range of agricultural chemicals and pesticides, contributing to the lake’s eutrophic and polluted status [5].

Despite the shallowness of the Salton Sea, it is a holomictic lake that experiences regular stratification in the warm summer months. Temperatures in the region rise and warm the surface water of the lake, creating a thermocline that separates the surface water (i.e., epilimnion) from the bottom waters (i.e., hypolimnion). The difference in density throughout the water column prevents dissolved oxygen in the epilimnion from flowing into to the hypolimnion, leading to an oxycline (i.e., an oxygen gradient). Anoxia in the hypolimnion allows for anaerobic, sulfate reducing bacteria to decompose organic matter and reduce sulfate (SO_4_^2−^) to hydrogen sulfide (H_2_S), leading to H_2_S accumulation in the hypolimnion [5, 6]. As temperatures in the area cool, the water column thermocline dissipates and lake turnover ensues, oxygenating the water column and stimulating sulfide oxidation. Lake mixing continues until temperatures in the Salton Sea region rise again in late spring, initiating the lake stratification cycle again. This seasonal stratification is expected to weaken as the lake continues to shrink because the lakes’ shallow depth will prevent an oxycline from forming, inhibiting sulfate reduction in an oxygenated water column and subsequently preventing the accumulation of H_2_S in the hypolimnion.

Seasonal lake stratification and oxidation–reduction regulate the lake’s sulfur cycle, which greatly impacts the health and stability of this ecosystem and its surrounding population. Summer winds in the region are occasionally strong enough to overcome the shallow lake’s stratification, causing upwellings that introduce H_2_S from the reducing hypolimnion to the oxic epilimnion [5]. Rapid sulfide oxidation consumes the available dissolved oxygen and contributes to gypsum (i.e., CaSO_4_) crystal formation and precipitation, covering the surface of the Salton Sea in what is known as a gypsum bloom [6, 7]. CaSO_4_ and MgSO_4_ minerals have been found in high concentrations in the Salton Sea’s exposed playa and dust attributed to the playa [8, 9]. Salton Sea dust has been shown to induce pulmonary inflammation and is associated with the respiratory distress experienced by the local population [10, 11]. Furthermore, Miao et al. (2024) recently found that particulate matter (i.e., PM) originating from the Salton Sea, especially during gypsum and/or algal bloom events, corresponds to increased hospitalizations locally [12]. Thus, the sulfur cycle in the Salton Sea is a crucial process that not only contributes to this ecosystem’s dynamic structure and function but impacts public health in the region.

While the Salton Sea’s seasonal stratification and sulfur cycle have been well studied, the involvement of microorganisms in these dynamic processes has been neglected. Microorganisms are integral players in biogeochemical cycling and serve as the interface between ecological and public health, and there is a need to understand how microorganisms are involved in the geochemical cycling of extreme, vulnerable ecosystems like the Salton Sea [13–15]. The ongoing reduction in the lake’s volume and surface area will continue to expose the playa to wind erosion, introducing remnants of the lake into the atmosphere [7, 13, 14]. The connection between the evaporites in the Salton Sea and its playa dust has been explored, yet the microbial constituents cycling these compounds are uncharacterized. Without investigating how the microbiome contributes to the biogeochemistry of the Salton Sea, we cannot holistically understand and mitigate the ecosystem’s degradation, which imminently threatens local public health. Here, we explored the taxonomic and functional diversity of the Salton Sea microbiome across seasons throughout the water column. We utilized amplicon, marker gene sequencing (i.e., 16S rRNA sequencing) to determine the microbial composition of the Salton Sea water columns, as well as shotgun metagenomic sequencing to assess the functional capacity of a subset of these samples, with special attention given to sulfur cycling metabolisms. The goal of this work is to understand the distribution of microorganisms and their functions within the Salton Sea across time, and how these microbial communities shape the geochemistry of their extreme, unstable environment.

## Methods

### Geochemical sampling methods

For all water monitoring and sampling, the deepest portion of the southern basin was accessed at 33° 15′ 45.54″, -115° 44′ 20.4″. Temperature, conductivity, pH, turbidity, dissolved oxygen (DO; in mg/L and percent saturation), dissolved organic matter (DOM), salinity, and oxidation–reduction potential (ORP) was determined in situ with a calibrated YSI EXO2 multi-parameter sonde probe (YSI Incorporated, Yellow Springs, OH, USA; Table 1). Water column samples were collected with a battery-powered peristaltic pump with in situ filtering capabilities (i.e., ALEXIS peristaltic pump, Proactive Environmental Products) from within a boat at the water surface. Samples were collected at the following eight depths: 0 m, 3 m, 4 m, 5 m, 7 m, 9 m, 10 m, and 10.5 m in August 2021, December 2021, and April 2022, with 10.5 m being the maximum depth (Table 1). The peristaltic pump hose was attached to the probe’s end such that water was sampled and monitored at discrete depths simultaneously. The hose was flushed for ~ 2 min between each collection. These sampling depths were selected at the chemoclines to access the H_2_S gradient throughout the water column. Water samples were stored in acid-washed 1L Nalgene bottles. Samples collected for H_2_S and SO_4_^2−^ analyses were filtered at 0.4 micron and preserved immediately with powdered zinc acetate for H_2_S and SO_4_^2−^ concentration determinations.

**Table 1:** Sample metadata and geochemistry data

Sample ID	Sample Month	Sample Year	Depth (m)	Local %DO	Dissolved Oxygen (mg/L)	Oxidative-Reduction Potential (mV)	Temperature (°C)	Salinity (ppt)	Dissolved Organic Matter (RFU)	Sulfate (mM)	Sulfide (μM)
8.24.21.0m	August	2021	0	74.6	3.88	0.8	31.150	57.00	29.46	180.56	10.51
8.24.21.3m	August	2021	3	37.8	1.99	65.2	30.434	57.18	30.02	181.55	3.15
8.24.21.4m	August	2021	4	23.9	1.26	65.3	30.369	57.23	30.08	181.12	13.98
8.24.21.5m	August	2021	5	16	0.84	64.5	30.315	57.23	30.2	183.48	2.58
8.24.21.7m	August	2021	7	11.8	0.62	61.9	30.268	57.31	30.28	178.14	2.99
8.24.21.9m	August	2021	9	0.8	0.04	-30	30.377	57.42	30.8	184.72	30.56
8.24.21.10m	August	2021	10	0.2	0.01	-240.9	30.448	57.54	31.19	184.59	55.95
8.24.21.10.5m	August	2021	10.5	0.2	0.01	-240.9	30.448	57.54	31.19	183.00	66.69
12.22.21.0m	December	2021	0	105.1	6.82	65.3	16.149	61.54	28.74	195.45	2.71
12.22.21.3m	December	2021	3	88.4	5.76	66	15.926	61.51	28.89	193.76	2.59
12.22.21.4m	December	2021	4	87.1	5.68	64.7	15.900	61.52	28.87	194.42	2.59
12.22.21.5m	December	2021	5	87.2	5.69	66.1	15.899	61.52	28.86	195.62	2.55
12.22.21.7m	December	2021	7	86.9	5.67	65.6	15.893	61.52	28.89	197.57	2.81
12.22.21.9m	December	2021	9	84.9	5.6	66.4	15.359	61.49	28.75	196.52	2.71
12.22.21.10m	December	2021	10	85.2	5.61	68.3	15.365	61.49	28.76	193.36	2.88
12.22.21.10.5m	December	2021	10.5	85.2	5.61	68.3	15.365	61.49	28.76	196.90	2.77
4.13.22.0m	April	2022	0	92.4	5.24	71.7	24.391	60.74	25.88	188.06	3.09
4.13.22.3m	April	2022	3	69.8	4.23	70.8	20.304	60.95	28.00	187.46	2.66
4.13.22.4m	April	2022	4	59.7	3.63	70.8	20.122	60.61	28.11	184.97	3.61
4.13.22.5m	April	2022	5	56	3.41	71	20.098	60.56	28.13	186.86	3.64
4.13.22.7m	April	2022	7	52.4	3.19	70.7	20.075	60.56	28.22	169.51	3.20
4.13.22.9m	April	2022	9	51.2	3.12	70.7	20.069	60.54	28.28	184.37	3.06
4.13.22.10m	April	2022	10	51	3.11	70.9	20.066	60.56	28.29	179.15	3.44
4.13.22.10.5m	April	2022	10.5	51	3.11	70.9	20.066	60.56	28.29	172.16	3.82

This table contains the sample metadata and geochemistry measurements (i.e., local % dissolved oxygen, dissolved oxygen (mg/L), oxidative-reduction potential (mV), temperature (°C), salinity (ppt), dissolved organic matter (relative fluorescence units; RFU), sulfate (milliM), and sulfide (microM) for each sample collected during August 2021, December 2021, and April 2022

Water column sulfates were precipitated as BaSO_4_ by addition of saturated BaCl_2_ solution (250 g/L) followed by brief acidification (4N HCl) to remove carbonates, rinsed to neutral pH and remove sodium chloride, and then dried. SO_4_^2−^concentrations were determined gravimetrically. Total dissolved sulfide (ΣS^2−^ = H_2_S + HS^−^ + S^2−^) concentration in the water column (i.e., liquid phase) were determined from 1 mL sample aliquots dispensed into 2 mL microcentrifuge tubes pre-filled with 0.5 mL of 20% zinc acetate solution as a measurement standard. Samples were then vortexed for 5 s and stored at 4 °C in the dark until analysis. Sulfide concentration was determined colorimetrically using the method of Cline [15].

### Seawater collection & processing

Upon collection, the 1L seawater samples were transported back to the lab on ice and immediately filtered through two subsequent vacuum filtrations. For the first filtration, an acid-washed, sterilized glass funnel holding an autoclaved 5 μm filter (47-mm diameter; Durapore Membrane filters, Millipore Sigma, Temecula, CA, USA) was used to filter the 1L sample into an acid-washed, 1L flask to remove large aggregates from the sample. The resulting filtrate is then immediately vacuum filtered through an acid-washed, sterilized glass funnel holding a sterile 0.2 μm filter (47-mm diameter; Pall Supor 200 Sterile Grid filters, Pall Corporation, Port Washington, NY, USA) into an acid-washed, 1L flask. This second filtration is performed to capture microbial biomass on the 0.2 μm filters for future DNA extractions. Multiple 0.2 μm filters were used to process each 1L sample. Both the 5 and 0.2 μm filters were stored in sterile Whirl–pak bags respectively at -20 °C.

### DNA extraction and amplification

DNA extraction from the 0.2 μm filters were performed in duplicate with the Qiagen DNeasy PowerWater kit (Qiagen, Germantown, MD, USA), and the extracts were quantified with a NanoDrop 2000 (Thermo Fisher Scientific, Wilmington, DE, USA). Half of the duplicate extracts were then purified via a bead clean-up using AMPure XP Beads and quantified with a NanoDrop 2000. Raw and clean DNA extracts were stored at − 20 °C. Clean DNA extracts were first amplified, then subsequently indexed (without amplification) via a 2-step PCR. Extracts from August 2021 were amplified with Nextera-adapted Klindworth primers [16] targeting the 16S rRNA V3-V4 region in the first round of PCR. The 16S rRNA V3-V4 region is a universal marker gene for identifying bacterial and archaeal taxa down to the species level [17]. Amplification products were cleaned in an AMPure magnetic bead clean up step then indexed with Illumina Nextera XT indices (Illumina, San Diego, CA, USA). Clean DNA extracts from December 2021 and April 2022 were quantified using the NanoDrop 2000 and high-yield samples (> 10 ng/uL) were normalized to 10 ng/uL. Clean and normalized samples were amplified with DipSeq adapted Klindworth primers [16] targeting the 16S rRNA V3-V4 region and cleaned up using an AMPure magnetic bead clean up step before being indexed using DipSeq indices. While the use of different sequencing indices may introduce potential variation, denoising and filtering of the reads via the DADA2 pipeline (please see the “Bioinformatics-Amplicon Sequence Data” section) yielded an even distribution of reads across samples before amplicon sequence variants (ASV) were assigned (Supplemental Fig. 1). Furthermore, raw sequencing reads were transformed and/or normalized before downstream analyses. For the August 2021 samples prepared with the Nextera XT Index Kit, each amplification reaction contained the following: 1 μL of DNA template, 5 μL each of the 1 μM forward and reverse index primers, 12.5 μL of PCR KAPA HiFi HotStart Ready Mix, and 1.5 μL of PCR grade water to create a 25 μL reaction. For the December 2021 and April 2022 samples prepared with the DIP-seq adapted Klindworth primers, each amplification reaction contained the following: 2 μL of DNA template, 12.5 μL Phusion HSII Hi-Fidelity Ready Mix, 1 μL each of the 1 μM forward and reverse index primers, 0.1 μL of BSA, and 8.5 μL of water to yield a 25 μL reaction. Before sequencing submission, indexed products were cleaned with an AMPure magnetic bead clean up step and quantified using Qubit. Samples were then pooled relative to their DNA concentration.

### DNA sequencing

The amplified, pooled DNA extracts were sequenced via the Illumina, Inc. MiSeq platform by the UC Riverside Genomics Core. Raw DNA extracts collected from the 0 m, 5 m, and 10 m-depth samples from each time point with a concentration of at least 20 ng/μL were sent on dry ice to the SeqCenter for shotgun metagenome sequencing. The SeqCenter prepared these libraries using the Illumina DNA Prep kit and IDT 10 bp UDI indices and sequenced the libraries on an Illumina NextSeq 2000 (2 × 151 bp).

### Bioinformatics – amplicon sequence data

Amplicon sequences were demultiplexed by the UC Riverside Genomics Core, and the FASTQ sequences were assessed for sequencing quality via FastQC [18]. In addition to FastQC, the eestats2 program [19] was used to determine the percentage of reads of specific lengths that will pass through the expect error threshold for a specific sample. The results supplied by FastQC and eestats2 were used to determine where the reads should be trimmed across the samples. Before trimming, there was a total of 4,237,100 reads across all 24 samples (including forward and reverse reads) that were 301 base pairs long. The reads were then trimmed and filtered with BBDuk, a k-mer-based trimming and decontamination program from the BBTools suite created by the Joint Genome Institute [20], resulting in a total of 4,232,018 trimmed reads across the samples. After trimming, the Divisive Amplicon Denoising Algorithm 2 (DADA2) pipeline [21] was used via the RStudio environment (version 2023.03.0 + 386) to assign reads to amplicon sequence variants (ASVs). Contaminant ASVs identified by the “decontam” package for R, as well as ASVs identified in the PCR positive and negative controls, were removed from the ASV count data. Singletons and ASVs that were assigned to “Chloroplast” or “Mitochondria” taxonomic classifications were also removed from the ASV count data set [22]. Prior to decontamination (i.e., removing ASVs identified in library preparation or sequencing controls, as well as ASVs assigned to mitochondria or chloroplasts), there were a total of 515,899 ASVs. After decontamination, there was 313,035 ASVs that were used for taxonomic identification.

### Bioinformatics – metagenome sequence data

In total there were nine samples submitted for shotgun metagenome sequencing, with each metagenome collected at the 0 m, 5 m, or 10 m depths in August 2021, December 2021, and April 2022. The sequence quality of the shotgun metagenomic data was assessed using FastQC [18], and adapter and primer sequences were trimmed using BBDuk [20]. There was a total of 126,416,634 read pairs before the metagenome sequences prior to trimming. After trimming, there were 120,169,462 read pairs used for contig assembly. BBNorm (BBTools suite, [20] was used to normalize the depth of trimmed read coverage in each metagenome. This normalization step ensures that there is an equal distribution of reads across all the sequenced regions, which is a necessary consideration with shotgun metagenomes due to their unequal sequence coverage [20]. The normalized reads are then error-corrected with SPades [23] and subsequently used for contig assembly with metaSPades [24], a metagenome-specific assembler available within SPades. The quality of the assembled contigs was determined using MetaQuast [25]. Trimmed, non-normalized metagenomic reads were then aligned to the assembled contigs using BWA-MEM. After read mapping, contigs and scaffolds were binned into genomes (i.e., metagenome-assembled genomes; MAGs) with metaBAT [26], using the read mapping results from BWA-MEM to guide the binning. The quality and completeness of the MAGs was determined using CheckM [27]. A custom bash script was then used to read the output from CheckM and parse out bins based on their completeness and contamination, identifying high-quality (i.e., > = 90% completeness, < 5% contamination) bins for downstream analyses ( [28]; Supplemental Table 2). Gene prediction was performed on the contigs and high-quality MAGs respectively using Prodigal [29]. KOFamScan was then used to assign functions and KEGG orthologies (i.e., KO identifiers) to the predicted genes [29, 30] in the contigs and high-quality MAGs. Genes assigned the same KO ID are functional orthologs of one another, and thus code for the same function across organisms. High-quality MAGs were also taxonomically annotated using GTDB-tk [31].

The number of reads that mapped to each gene in both contigs and high-quality MAGs was determined using featureCounts [32], which compares the alignment file created by BWA-MEM and the predicted genes found by Prodigal. The number of reads mapped to each gene calculated by featureCounts was combined with the functional annotations from KOFamScan via a custom bash script. The featureCounts results were then used to calculate depth of coverage for each gene in R by dividing the number of reads mapped to a gene by the gene’s length. Multiple genes were assigned the same KO identifier(s); thus, the coverage for each gene assigned the same KO were summed together to calculate coverage per KO assignment within contigs and high-quality MAGs respectively. The summed depth of coverage per KO was subsequently transformed via a center-log ratio transformation using the vegan package’s “decostand” command [33] to normalize the gene coverages by their respective sample library size [34–37]. Non-transformed, summed KO coverages within the contigs and high-quality MAGs were used as input in a custom R script to create binary presence-absence tables for functions of interest.

### Statistical analyses and data visualization

The 16S rRNA amplicon data, the annotated contigs and MAGs, and the geochemistry data (i.e., dissolved oxygen and percent saturation of dissolved oxygen (%DO), oxidative-reduction potential (ORP), dissolved organic matter (DOM), salinity, temperature, SO_4_^2−^ concentrations, and H_2_S concentrations) were analyzed in the RStudio environment using R software version 4.2.2. All environmental variables considered were centered and scaled via the “scale” function from “base” package in R before statistical analyses were performed. Correlations between the environmental variables were determined using the “cor.test” function from the “stats” package and visualized using the “corrplot.mixed” function from the “corrplot” package [38].

Raw, decontaminated 16S V3-V4 rRNA amplicon counts were rarefied to a sequencing depth of 7,381 using the “rrarefy” function from the “vegan” package [33]. The sequencing depth used for rarefaction was the minimum number of total ASV counts observed across samples, which was identified using the “min” and “rowSums” functions from the “base” package in R. Shannon-Weiner diversity and species richness (i.e., alpha diversity) of the rarefied 16S V3-V4 rRNA amplicon count data (i.e., microbial composition data) were calculated using the “diversity” and “specnumber” functions from the “vegan” package. Shannon-Weiner diversity and species richness were assessed for normality via Shapiro-Wilks tests using the “shapiro.test” function from the “stats” package. The Shapiro-Wilks test determined that Shannon-Weiner diversity was normally distributed (*P* = 0.711) and species richness was not normally distributed (*P* = 0.009), and thus, analyzing species richness would require non-parametric statistical tests for this work. A t-test was used to compare the means of Shannon-Weiner diversity between time points by using the “t.test” function from the “stats” package, and *p*-values were adjusted using the Bonferroni correction via the “p.adjust” function from the “stats” package. An analysis of variance (ANOVA) was also used to compare the variance of Shannon-Weiner diversity between time points by using the “aov” function from the “stats” package. After the ANOVA, a post hoc Tukey’s Honest Significant Difference (HSD) test was used via the “TukeyHSD” function from the “stats” package to determine which time points’ variances were significantly different from one another. Then, a Levene’s test was used via the “leveneTest” function from the “car” package to compare the homogeneity of variances across time points [39].

A Wilcoxon test was used to compare the mean of species richness between time points using the “wilcox.test” function from the “stats” package, and *p*-values were adjusted using the Bonferroni correction via the “p.adjust” function from the “stats” package. Additionally, a Kruskal–Wallis test was used to compare variance of species richness between time points using the “kruskal.test” function from the “stats” package. After the Kruskal–Wallis, a Dunn test was used via the “dunn_test” function from the “rstatix” package to determine which time points’ variances were significantly different from one another. To then compare the homogeneity of variances across time points, a Fligner-Killeen test using the “fligner.test” function from the “stats” package was performed.

To determine if environmental variables could accurately predict the distribution of Shannon-Weiner diversity and species richness across samples, generalized linear models were used. Specifically, to assess the impact of environmental variables on Shannon-Weiner diversity, a generalized linear model (i.e., GLM, a using a Gaussian distribution) was run via the “glm” function from the “stats”. As for species richness, a GLM (using a negative binomial distribution) was run via the “glm.nb” function from the “MASS” package. Environmental variables were chosen for these GLMs based on their ecological importance and on their correlations to one another. *P*-values from multivariate GLMs as well as the ANOVA and Kruskal–Wallis tests were adjusted using the Bonferroni correction via the “p.adjust” function from the “stats” package [40].

Beta diversity of the microbial composition data was performed by first transforming the data via a center-log ratio (i.e., CLR) transformation using the “decostand” function from the “vegan” package. This function adds a pseudo-count of 1 to all function counts, including those functions that have a count of zero, before performing the transformation [41]. Then a Euclidean distance matrix of the CLR-transformed 16S V3-V4 amplicon count data was created using the “dist” function from the “vegan” package and used as input to create a Principal Coordinates Analysis (i.e., PCoA) with the “pcoa” function from the “vegan” package. Homogeneity of variance in the microbial composition data across time points were compared using the “betadisper” function from the “vegan” package. Permutational multivariate analyses of variance (PERMANOVA) were performed with the “adonis2” function from the “vegan” package to determine if there were significant differences in microbial composition across time points and depths. All *p*-values for the PERMANOVAs were adjusted using the Bonferroni correction via the “p.adjust” function from the “stats” package.

A Detrended Correspondence Analysis (i.e., DCA) was performed using the “decorana” function from the “vegan” package to determine if there was an arch effect present within the microbial composition data across sites and within sites. Due to the length of the first DCA axes, Redundancy Analysis (i.e., RDA) was chosen to determine if and how the microbial composition data are constrained by the geochemistry data. RDAs were calculated using the “rda” function from the “vegan” package. The variation explained by the RDAs was obtained using the “RsquareAdj” function from the “vegan” package, and the significance of the RDAs was determined using the “anova” function from the “stats” package. The variance inflation factors for each predictor variable (i.e., the geochemistry data) in the RDAs was determined using the “vif.cca” function from the “vegan” package. To find the best fitting model, the “ordistep” and “ordiR2step” functions from the “vegan” package were used. The “ordistep” function builds the RDA stepwise to determine which variables lead to significant changes in variance and a lower AIC value for the model. The “ordiR2step” function builds the RDA stepwise based on which variables maximize the adjusted variation explained by each predictor variable considered (i.e., their adjusted R^2^) and are statistically significant. All *p*-values for the multivariate RDAs with the best fit were adjusted using the Bonferroni correction via the “p.adjust” function from the “stats” package.

To compare the variance of relative gene coverages by pathway or process across the metagenomes, the summed depth of gene coverages per KO, per pathway/process was transformed via a CLR transformation using the vegan package’s “decostand” command [33] to normalize the total pathway coverages by their respective sample library size [34–37]. Genes involved in thiosulfate oxidation, hydrogen sulfide oxidation, and sulfite oxidation were collectively labeled as “sulfur oxidation” for the purpose of this analysis. Kruskal–Wallis tests was used to compare the variance of the sulfur cycling pathways by time point, using the CLR-transformed, total KO coverages as input. Kruskal–Wallis tests were only performed on pathways or processes that contained at least two or more KOs considered in this work. After the Kruskal–Wallis, a Dunn test was used via the “dunn_test” function from the “rstatix” package to determine which time points’ variances were significantly different from one another. To then compare the homogeneity of variances across time points, a Fligner-Killeen test using the “fligner.test” function from the “stats” package was performed.

## Results

### Seasonal environmental differences

Environmental conditions throughout the water columns significantly varied across time, between August 2021, December 2021, and April 2022. Specifically, percent saturation of dissolved oxygen (i.e., %DO; *P* = 2.937e-04), dissolved organic matter (i.e., DOM; *P* = 3.508e-05), oxidative-reduction potential (i.e., ORP; *P* = 5.112e-05), sulfate concentration (i.e., SO_4_^2−^, *P* = 2.83e-06), hydrogen sulfide concentration (i.e., H_2_S, *P* = 0.005), and temperature (°C*,P* = 3.524e-05) significantly varied across sampling dates. H_2_S concentration was significantly different in August when compared to December (*P* = 0.03) and April (*P* = 0.016), whereas December and April did not significantly differ from one another (*P* = 0.703).

**Fig. 1:**
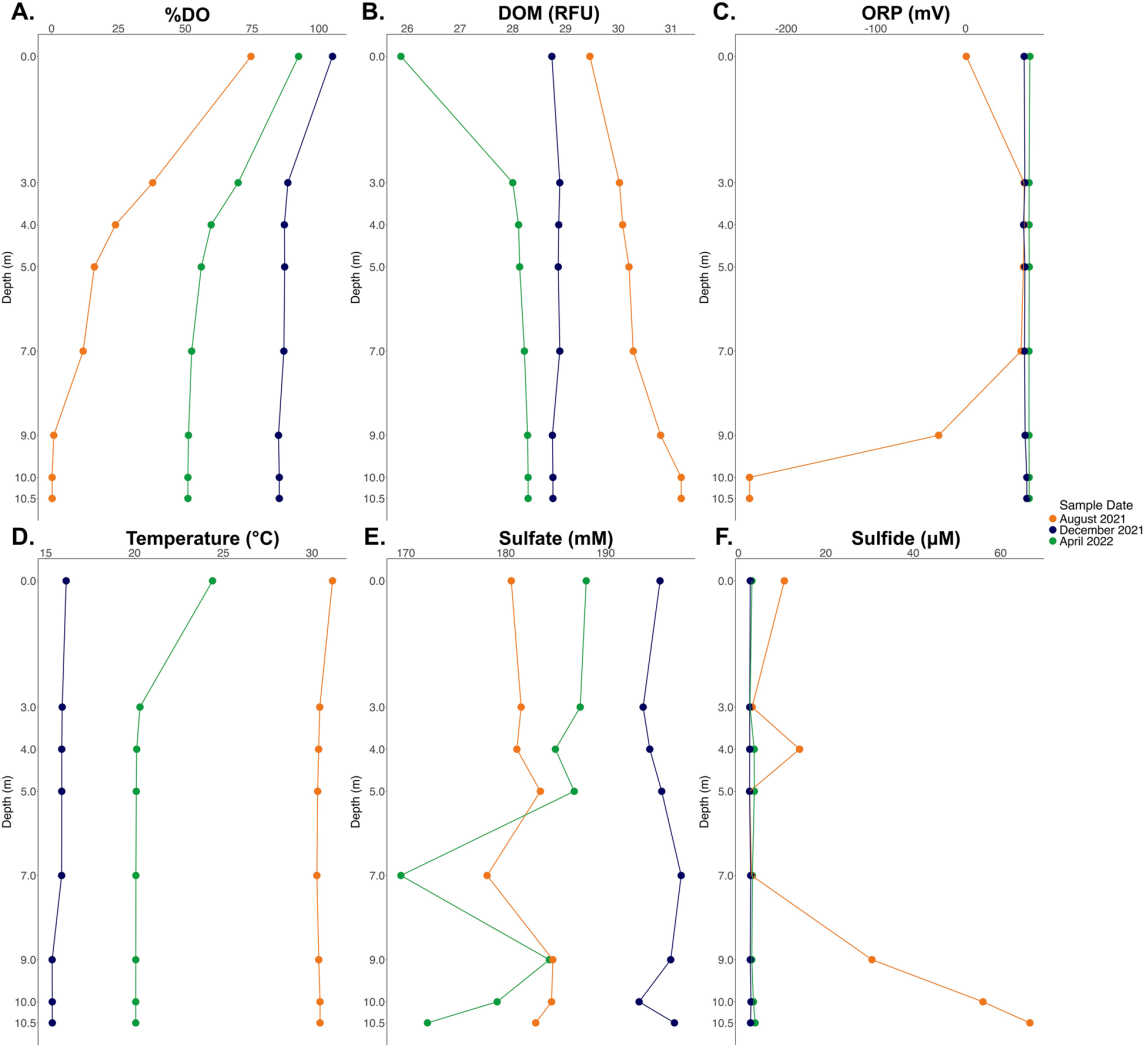
Vertical Profiles of (**A**) % Dissolved Oxygen, (**B**) Dissolved Organic Matter, (**C**) Oxidative-Reduction Potential, (**D**) Temperature, (**E**) Sulfate, and (**F**) Sulfide in Our Sampling Location in the Salton Sea. Measurements were taken in August 2021 (orange), December 2021 (blue), and April 2022 (green) at 8 different depths (0 m, 3 m, 4 m, 5 m, 7 m, 9 m, 10 m, and 10.5 m)

Salinity in the Salton Sea fluctuated (57 ppt – 61.54 ppt) between across August 2021, December 2021, and April 2022, reaching a high of 61.54 ppt in December 2021 (Table 1). Temperature was at its peak throughout the water column in August 2021 compared to December 2021 and April 2022, ranging from 30.27 – 31.15 °C. H_2_S concentration (~ 2.58 – ~ 66.69 μM) and DOM (29.46 – 31.19 relative fluorescence units; RFU) were also at their highest in August relative to December and April, particularly at 7 m and below. H_2_S reached a peak concentration of 66.69 μM in August 2021 at 10.5 m (Table 1). Conversely, ORP and %DO were at their lowest in August. DO had a concentration of 0.01 mg/L at 10 m in August 2021, corresponding to a %DO of 0.2 (Table 1). ORP reached -240.9 mV at 10 m in August 2021 (Table 1). By December 2021, temperature (15.37 – 16.15 °C) and H_2_S concentration (~ 2.55 - ~ 2.88 μM) throughout the water column were at their lowest whereas SO_4_^2−^ concentration (~ 188.06 - ~ 197.57 mM) and %DO (84.9% -105.1%) was at its highest relative to August and April. In April 2022, DOM (25.88 – 28.29 RFU) had reached its lowest concentration relative to August and December, whereas ORP (70.7 – 71.7 mV) was at its highest throughout the water column (Fig. 1).

### Alpha diversity

**Fig. 2:**
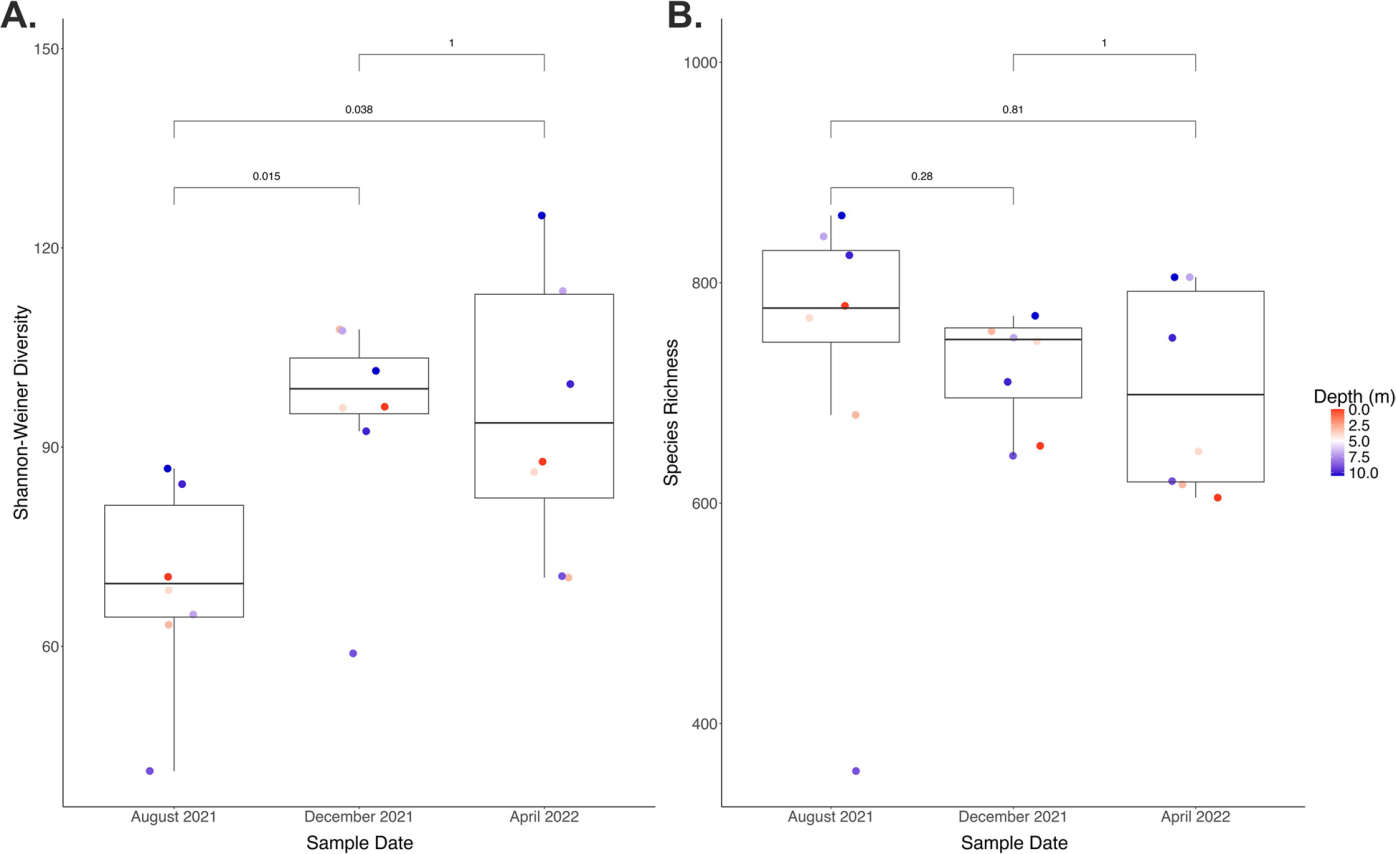
Shannon-Weiner diversity (**A**) and Species Richness (**B**) by Time Point. These box-and-whisker plots compare Shannon-Weiner diversity and species richness by time point. Each point represents a sample and are colorized by depth, with red being the shallow depths and blue as the deeper depths. The statistical comparisons shown are the adjusted *p*-values from a t-test comparing Shannon-Weiner diversity by time point and a Wilcoxon test for comparing species richness by time point

Shannon-Weiner diversity increased from August 2021 to December 2021 (Fig. 2, Supplemental Table 2). Mean Shannon-Weiner diversity in August 2021 was significantly lower than both December 2021 (*P* = 0.018) and April 2022 (*P* = 0.044), but December and April did not significantly differ from one another (*P* = 1). Additionally, the sample from the 9 m depth consistently had the lowest Shannon-Weiner diversity of all the samples from each time point (Supplementary Fig. 2). Shannon-Weiner diversity significantly varied across time points (*P* = 0.011), yet this trend did not hold true when comparing time points in a pair-wise fashion (Supplemental Table 3). A post hoc Tukey test revealed that the Shannon-Weiner diversity of August 2021 was significantly different than December 2021 (*P* = 0.017) and April 2022 (*P* = 0.01; Supplemental Table 3). However, Shannon-Weiner diversity did not significantly differ between December 2021 and April 2022 (*P* = 0.96; Supplemental Table 3). Lastly, a generalized linear model determined that temperature (°C, *P* = 0.0004) and SO_4_^2−^ (*P* = 0.036) together were the only environmental variables that significantly predicted Shannon-Weiner diversity (R^2^_*adj*_ = 0.406, *P* = 0.0016).

In contrast to Shannon-Weiner diversity, species richness appeared to slightly decrease across time points (Supplemental Table 2); but richness was not significantly different between any of the time points (Fig. 2). The variance of species richness also did not vary significantly overall (*P* = 0.5, Supplemental Table 3). However, as was observed with Shannon-Weiner diversity, the 9 m-depth sample at each time point consistently maintained the lowest species richness (Supplemental Fig. 2). Furthermore, ORP (*P* = 0.627) and SO_4_^2−^ concentration (*P* = 0.0085) interacted significantly to predict species richness across time points (McFadden’s Pseudo R^2^ = 0.346, *P* = 0.0057).

### Microbial composition and diversity

**Fig. 3:**
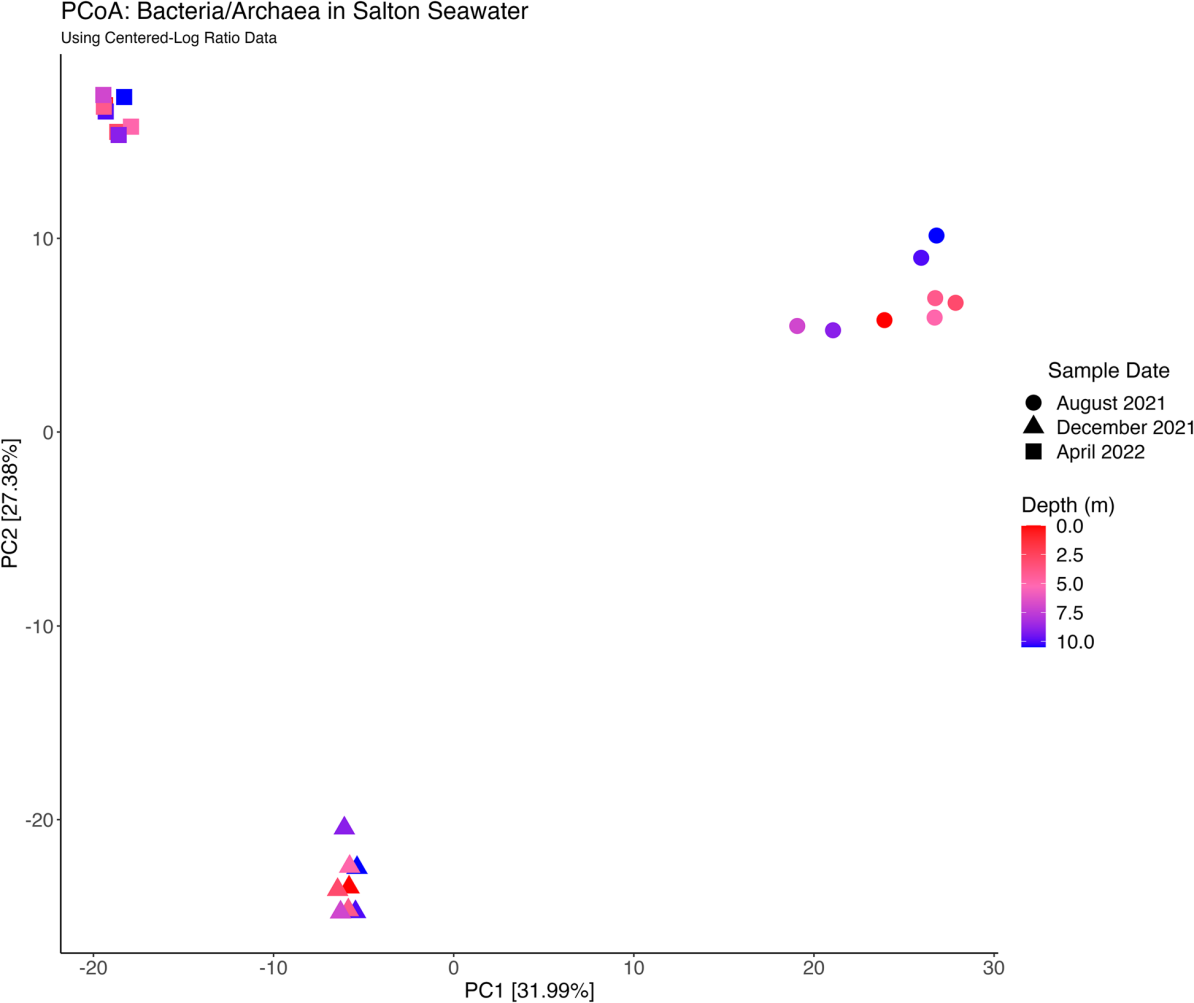
Principal Coordinates Analysis (PCoA) of Microbial Composition by Time Point and Depth. Each point represents a sample, with its shape corresponding to the sampling time point and the color corresponding to the sampling depth. The first axis of variation (PC1) represents 31.99% of the variation, and the second axis of variation (PC2) represents 27.38% of the variation

Microbial composition significantly varied between time points (*R*^2^ = 0.59, *P* = 0.003); however, the dispersion (i.e., spread) of these data was not equivalent across time points (*P* = 0.0003) and may contribute to these significant compositional differences. A principal coordinates analysis (PCoA) showed that microbial composition was tightly clustered by time point, rather than the depth that each sample originated from (Fig. 3). Microbial composition throughout the water column in August 2021 exhibited the greatest dispersion in microbial composition and was significantly greater than the microbial dispersion in December 2021 (*P* = 0.0034) and April 2022 (*P* = 0.0004). A PERMANOVA confirmed that the variance in microbial composition across time points were significantly different from one another: August 2021 and December 2021 (*R*^2^ = 0.49, *P* = 0.003), December 2021 and April 2022 (*R*^2^ = 0.55, *P* = 0.003), and August 2021 and April 2022 (*R*^2^ = 0.52, *P* = 0.006; Supplemental Table 4).

Within each time point, patterns of non-major microbial taxa (i.e., bacterial genera with a relative abundance of < 1%) remained consistent throughout the water column (Supplemental Fig. 3). Thus, we describe the total relative abundance of major microbial taxa throughout the water column by time point as opposed to focusing on specific depths. Two microbial families, *Microbacteriaceae* and *Nitriliruptoraceae*, dominated the water column microbiome across depths and time points. The relative abundance of *Microbacteriaceae* decreased from 29.45% in August 2021 to 27.29% in December 2021 to 11.49% by April 2022. Conversely the relative abundance of *Nitriliruptoraceae* increased from 12.18% in August 2021 to 14.08% in December 2021 to 26.98% in April 2022. *Litoricolaceae* appeared as the third most abundant family in August 2021 (10.24%) and April 2022 (9.24%) but was replaced by *Ilumatobacteraceae* in December 2021 (9.98%).

**Fig. 4:**
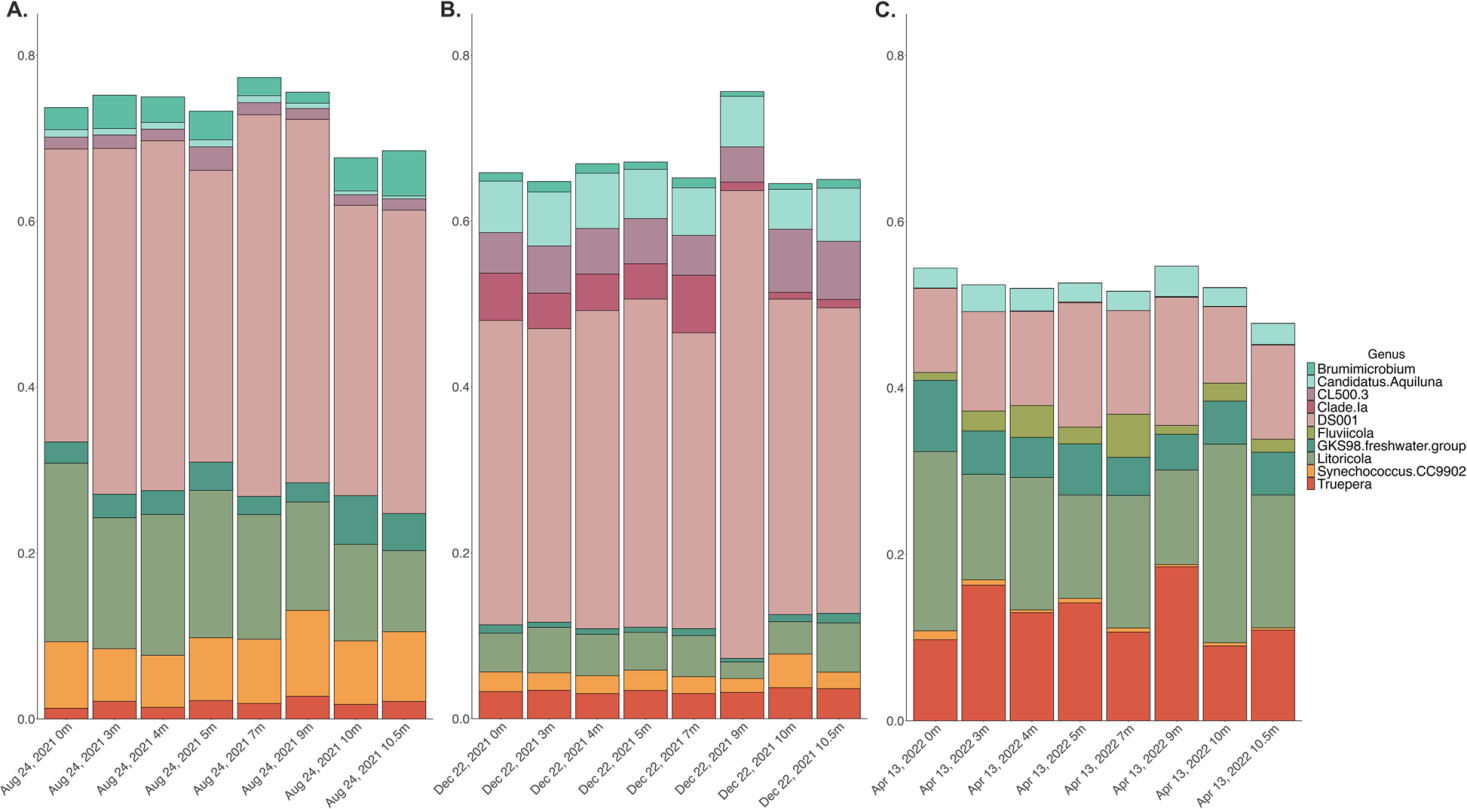
Relative Abundance of the Top 10 Most Abundant Bacterial Genera by Time Point. These stacked bar plots display the relative abundance of the top 10 most abundant bacterial genera (16S rRNA) in August 2021 (**A**), December 2021 (**B**), and April 2022 (**C**). The x-axis contains the samples organized in order by date and increasing depth, and the y-axis is the relative abundance

A single genus from the *Microbacteriaceae* family, DS001, accounted for over a third of the total relative abundance of the water column microbiome in August 2021 (39.40%) and December 2021 (39.57%; Fig. 4). By April 2022, the relative abundance of DS001 throughout the water column had dropped to 11.87%. *Litoricola* represented 15.22% of the relative abundance throughout the water column in August 2021, decreased to 4.51% of the total relative abundance in December 2021, then increased to 16.66% of the water column microbiome in April 2022. *Synechococcus* CC9902 was the third most abundant genus in August 2021 at 7.78% of the total relative abundance, but decreased to 2.44% in December 2021, then to less than 1% in April 2022. *Truepera* increased in total relative abundance from 1.96% in August 2021 to 3.36% to 12.56% in April 2022, becoming the second most abundant genus in the water column in April 2022.

**Fig. 5:**
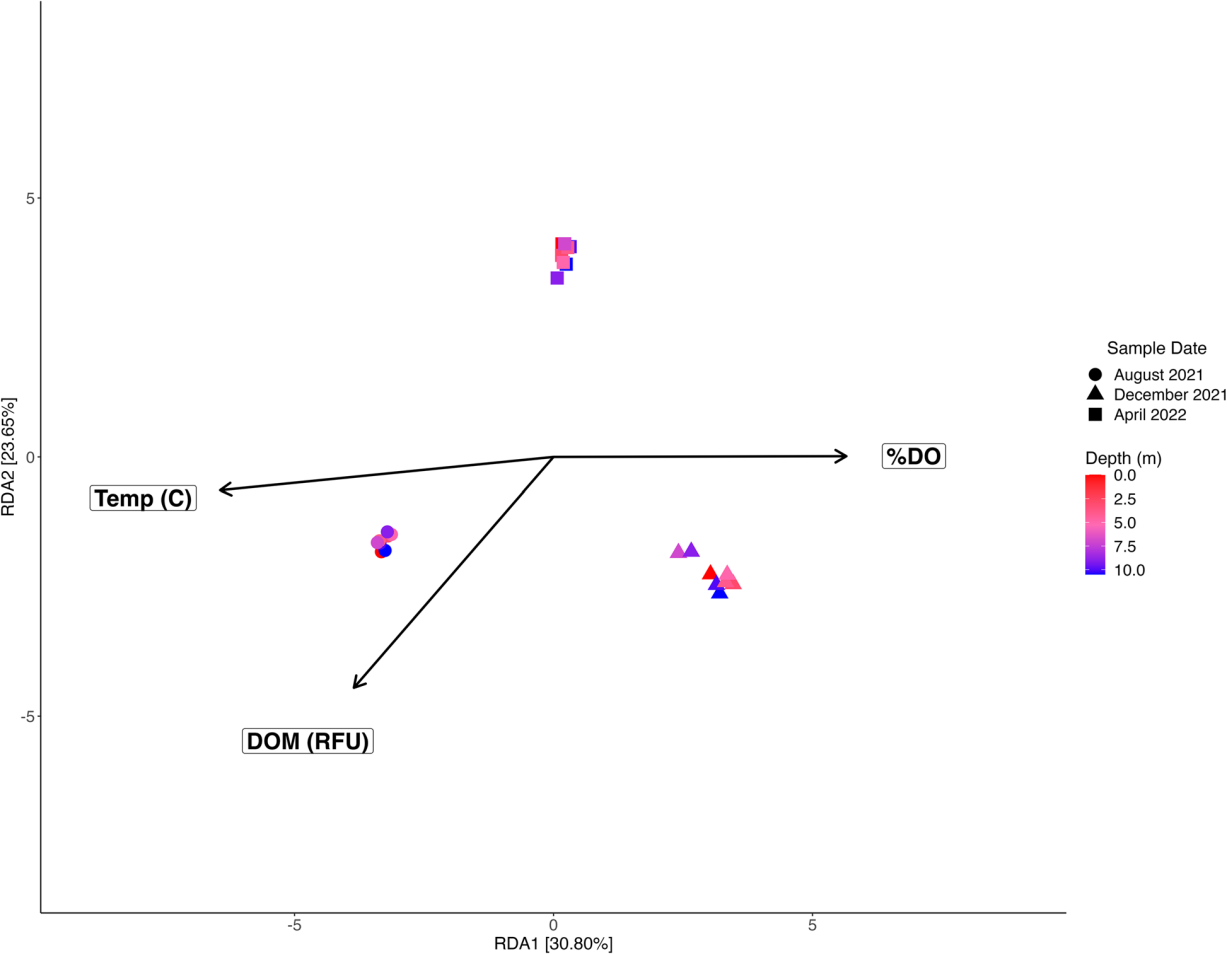
Redundancy Analysis of Environmental Variables and Microbial Composition. This is a redundancy analysis showing the significant environmental variables driving microbial (16S rRNA) composition. Each point represents a sample with its shape corresponding to its sampling time point, and the color corresponding to its sampling depth. Temperature (*R*^2^ = 0.27, *P* = 0.004), dissolved organic matter (DOM; *R*^2^ = 0.13, *P* = 0.004), and percent saturation of dissolved oxygen (%DO; *R*^2^ = 0.089, *P* = 0.004) significantly drive microbial composition across sites

Temperature, DOM, and %DO were significant environmental drivers of overall microbial composition throughout the water column (Fig. 5, Supplemental Table 5). Redundancy analyses found that temperature was the environmental driver that explained the greatest variance in microbial composition across time points (*R*^2^ = 0.27, *P*_*adj*_ = 0.004), followed by DOM (*R*^2^ = 0.13, *P*_*adj*_ = 0.004), then %DO (*R*^2^ = 0.089, *P*_*adj*_ = 0.004). When evaluating environmental drivers of microbial composition within August 2021, DOM appeared to be the only significant variable (*P*_*adj*_ = 0.002). However, DOM only explained 10.91% of the variation observed in the microbial assembly in this time point. In December 2021, ORP was the most significant environmental driver (*P*_*adj*_ = 0.012), but only explained 4.23% of the total variation in microbial composition. DOM was the only near significant environmental driver in April 2022 (*P*_*adj*_ = 0.068) but constrained only 1.5% of the total variation in microbial composition.

### Taxonomic annotation of MAGs

A total of 1,907,889 contigs were assembled from these nine metagenomes and subsequently binned into 231 metagenome-assembled genome (MAG) bins. Bins with 90% and < 5% contamination were classified as “good” bins and used for taxonomic annotation [42, 28]. Out of 231 MAGs, 19 bins had a completeness of > 90% and contamination < 5% and thus were selected for functional and taxonomic annotation. All 19 MAGs were identified as *Bacteria* and were assigned to the following phyla: *Proteobacteria* (*n* = 13), *Actinobacteriota* (*n* = 4), *Bacteroidota* (*n* = 2); Supplemental Table 6). Eight MAGs were assigned to the genus HIMB30 of the *Litoricolaceae* family, and were retrieved from metagenomes at all depths and time points except for the 5 m-depth August 2021 sample. Three MAGs were assigned to the genus Casp-actino5 of the family *Ilumatobacteraceae*. One MAG was assigned to the SKUL01 genus of the *Cryomorphaceae* family and another MAG was assigned to M55B157 genus in the S36-B12 family.

### Functional annotation of metagenomes

Gene coverage as well as the presence/absence of genes of interest were used to determine how prevalent certain functions of interest are in both contigs and MAGs throughout the water column. To understand the nutritional strategies employed by microorganisms in the Salton Sea, genes involved in sulfur energy metabolisms, phototrophy, and carbon fixation pathways were examined in the contigs and MAGs respectively.

#### Sulfur cycling genes

KO identifiers of genes involved in assimilatory sulfate reduction, dissimilatory sulfate reduction, thiosulfate oxidation, hydrogen sulfide oxidation, sulfite oxidation, and sulfur disproportionation were compared within and between each sample metagenome’s contigs.

The relative depth of coverage of specific sulfur cycling pathways and processes varied across all depths and time points. Sulfur oxidation significantly varied by time point (*P* = 0.027). Here, sulfur oxidation included genes in the thiosulfate oxidation pathway (i.e., *soxABCDXYZ*; the SOX pathway), hydrogen sulfide oxidation genes (i.e., hydrogen sulfide:quinone oxidoreductase, *sqr*; hydrogen sulfide dehydrogenase flavoprotein chain, *fccB*), and the sulfite oxidation gene sulfite dehydrogenase (quinone) subunit SoeA (i.e., *soeA*). The dissimilatory sulfate reduction/reverse dissimilatory sulfate reduction pathway (i.e., dissimilatory sulfite reductase subunits alpha and beta, *dsrAB*; adenylylsulfate reductase, subunits A and B, *aprAB*) also significantly varied by time point (*P* = 0.039). Conversely, the assimilatory sulfate reduction pathway did not significantly vary by time point (*P* = 0.058). This pathway contained the sulfite reductase (i.e., ferrodoxin, *sir*), sulfate adenylyltransferase subunit 2 (i.e., *cysD*), phosphoadenosine phosphosulfate reductase (i.e., *cysH*), bifunctional enzyme CysN/CysC (i.e., *cysNC*), sulfite reductase (NADPHD) hemoprotein beta-component (i.e., *cysI*), and sulfite reducatse (NADPH) flavoprotein alpha-component (i.e., *cysJ*). These genes and their respective functions are described in depth below.

##### Sulfur oxidation and disproportionation genes

**Fig. 6:**
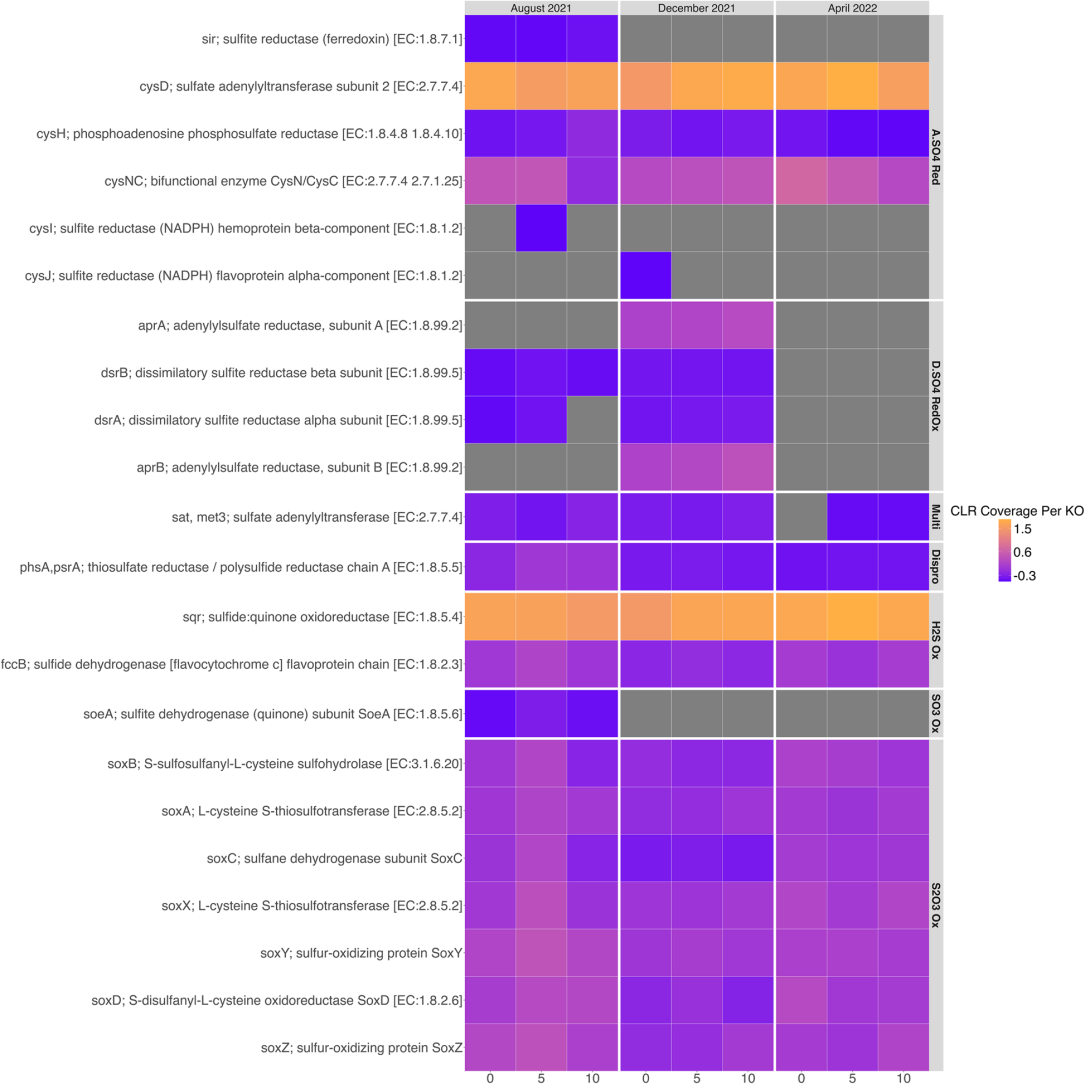
Relative Coverage of Sulfur Metabolic Genes in Salton Seawater Metagenomes. This is a heatmap detailing the relative coverage (i.e., centered-log ratio transformation of read coverage per gene) of sulfur cycling genes identified in the 0 m, 5 m, and 10 m-depth metagenomes. Each column represents a metagenome, which are sectioned by their respective sampling time points, and each row represents a different KO assignment given to genes found in the metagenomes. The darker purple the square, the higher the relative coverage of that KO is. Gray squares represent genes that were not found in the metagenomes

Genes involved in the SOX pathway exhibited relatively higher coverages in August 2021 compared to other sulfur cycling pathways in this time point (Fig. 6). The thiosulfate oxidation pathway can oxidize thiosulfate (S_2_O_3_^2−^), H_2_S, elemental S, and SO_3_^2−^ into SO_4_^2−^ [43, 44]. Depth of coverage was also relatively high for genes involved in oxidizing H_2_S to polysulfide species and/or elemental sulfur (i.e., *sqr*, *fccB*), as well as genes involved in sulfur disproportionation (i.e., thiosulfate reductase/polyhydrogen sulfide reductase chain, *phsA/psrA*; Fig. 6). Sulfur disproportionation is a process in which sulfur species act as an electron donor and an electron acceptor, yielding H_2_S and SO_4_^2−^. The SOX pathway, *fccB,* and *phsA/psrA* displayed the highest relative coverages in the 5 m-depth metagenome, whereas *sqr* had the highest relative coverage in the 0 m-depth metagenome. *soeA*, which oxidizes sulfite (i.e., SO_3_^2−^) to SO_4_^2−^, had lower relative coverage compared to other H_2_S oxidizing genes in August 2021, and was only observed in the August 2021 metagenomes. In December 2021, the 0 m, 5 m, and 10 m-depth metagenomes exhibited lower relative coverage of the SOX pathway and *fccB* compared to August 2021. *sqr* maintained high relative coverage across depths in December 2021, with the highest coverage also found in the 10 m-depth contigs. Relative coverage of genes in the SOX pathway and hydrogen sulfide oxidation (i.e., *sqr*, *fccB*) increased across the metagenomes in April 2022. *sqr* exhibited the highest relative coverage in the April 2022 metagenomes, with the highest coverage found in the 5 m-depth contigs.

##### Dissimilatory sulfate reduction/ reverse dissimilatory sulfate reduction

Two genes involved in dissimilatory sulfate reduction and reverse dissimilatory sulfate reduction (Dsr/rDSR*)*, *dsrAB,* were found in the August 2021 with low relative coverage (Fig. 6). *dsrAB* can oxidize H_2_S into SO_3_^2−^ via reverse dissimilatory sulfate reduction in aerobic conditions. Sulfate adenylyltransferase (i.e., *sat*, *met3*), which is involved in both dissimilatory and assimilatory sulfate reduction, exhibited a lower relative coverage in August 2021 compared to other sulfate reduction genes. *aprAB* and *dsrAB* had the highest relative coverage in December 2021 at all depths compared to other sulfur metabolic pathways at this time point. Specifically, *aprAB* had higher relative coverage than *dsrAB* across depths, with the highest relative coverage of *aprAB* in the 10 m-depth contigs. *aprAB* were only observed in the December 2021 metagenomes. *aprAB* can oxidize SO_3_^2−^ into adenylyl sulfate (APS). Additionally, relative coverage of *dsrAB* was higher in December 2021 than August 2021 at all sampling depths. Only one gene associated with the dissimilatory sulfate reduction/reverse dissimilatory sulfate reduction pathway, *sat/met3*, was found with low relative coverage in the April metagenomes from the 5 m and 10 m depths.

##### Assimilatory sulfate reduction

The assimilatory sulfate reduction pathway was almost complete in the August 2021 metagenomes, only missing *cysJ* across all depths and *cysI* at the 0 m and 10 m depths (Fig. 6). Two genes within this pathway, *cysNC* and *cysD*, exhibited relatively higher coverage in August 2021 compared to other assimilatory and dissimilatory sulfate reduction genes. *cysD* exhibited the highest coverage in the 0 m-depth metagenome in August 2021, and *cysNC* maintained similar relative coverage in the 0 m-depth and 5 m-depth metagenomes but decreased in the 10 m-depth metagenome. Two genes belonging to the assimilatory sulfate reduction pathway were observed only in August 2021: *cysI* in the 5 m-depth metagenome and *sir* found in each metagenome.

As was observed in August 2021, *cysNC* and *cysD* exhibited higher relative coverage across the depths in December 2021 compared to other KOs involved in the assimilatory sulfate reduction pathway. Both *cysD*, which reduces SO_4_^2−^ into APS, and *cysNC*, which reduces APS to phosphoadenylyl sulfate (PAPS), were observed to have the highest relative coverage in the 10 m-depth metagenome. The only observance of *cysJ*, a member of the assimilatory sulfate reduction pathway that reduces SO_3_^2−^ into H_2_S, was in the 0 m-depth metagenome in December 2021. High relative coverages of *cysD* and *cysNC* were maintained in April 2022. Specifically, *cysNC* exhibited the highest relative coverage in the 0 m-depth metagenome, and *cysD* exhibited the highest relative coverage in the 5 m-depth metagenome.

#### Sulfur cycling in MAGs

**Fig. 7:**
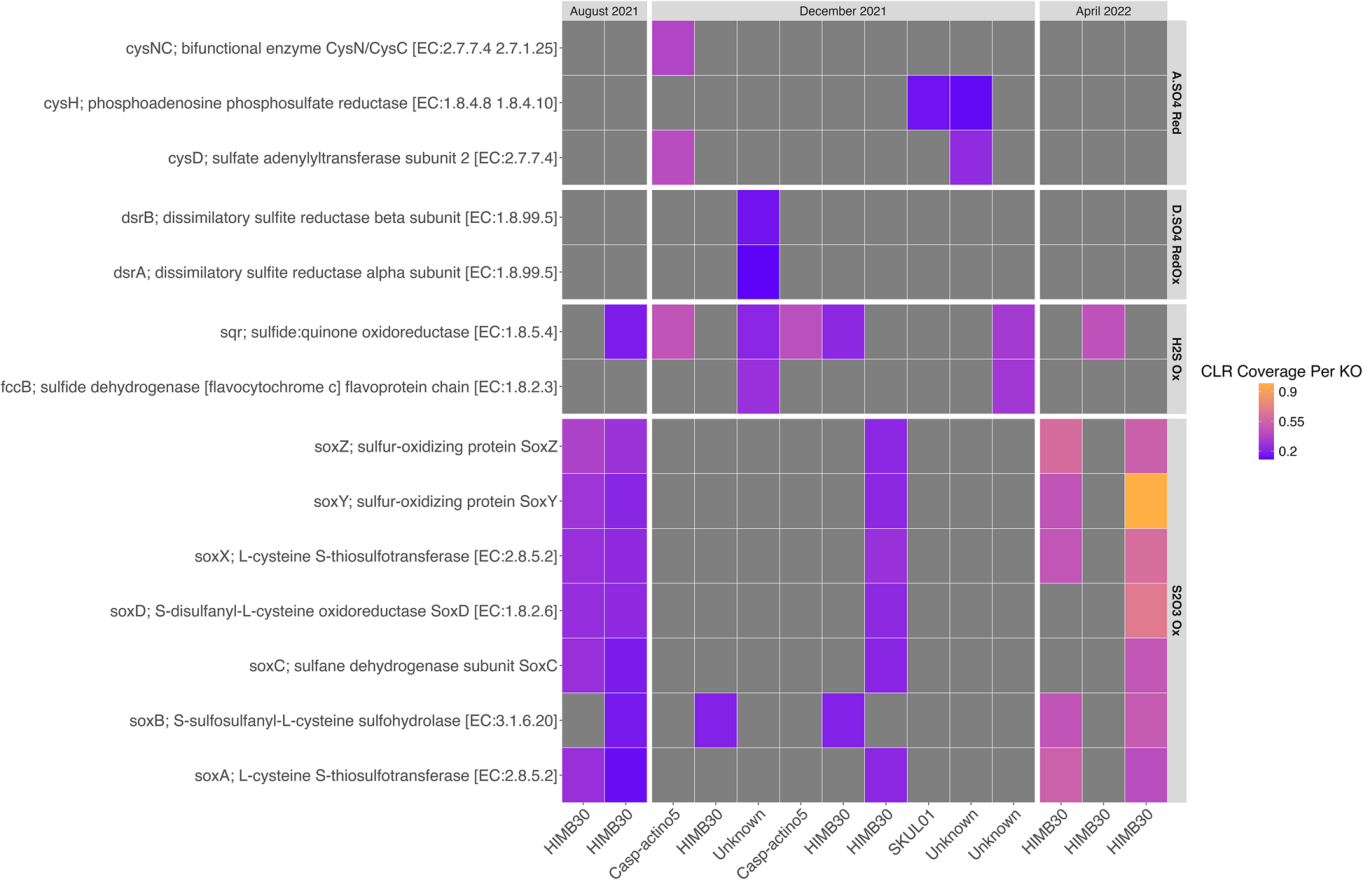
Relative Coverage of Sulfur Cycling Genes in Salton Seawater MAGs. This is a heatmap detailing the relative coverage (i.e., centered-log ratio transformation of read coverage per gene) of sulfur cycling genes identified in metagenome-assembled genomes (MAGs) found in the 0 m, 5 m, and 10 m-depth metagenomes. Each column represents a bin (i.e., MAG) and its associated genus assignment, which are separated by their respective sampling time points, and each row represents a different KO assignment given to genes found in the metagenomes. The darker purple the square, the higher the relative coverage of that KO is. Gray squares represent genes that were not found in the MAGs

KO identifiers of genes involved in the sulfur metabolisms described above were also examined within the MAGs from each sample. Of the 19 high-quality MAGs we identified and annotated, 12 MAGs contained sulfur oxidation and/or dissimilatory sulfate reduction genes (Fig. 7). Within these 12 MAGs, eight of the MAGs were assigned to the bacterial genus HIMB30. Two of the 12 MAGs were assigned to the bacterial genus Casp-actino5. Two MAGs contained only assimilatory sulfate reduction genes, and one of these MAGs was assigned to the genus SKUL01.

##### Sulfur oxidation and disproportionation genes

Two MAGs found in the 0 m-depth and 10 m-depth August 2021 samples respectively contained SOX genes (Fig. 7). The MAG found in the 0 m-depth August 2021 sample contained all the SOX genes excluding the *soxB* gene, whereas the MAG found in the 10 m-depth August 2021 sample contained all SOX genes (*soxABCDXYZ)* as well as the *sqr* gene involved in oxidizing H_2_S. These two MAGs were both assigned to the HIMB30 genus. Of the nine MAGs found in the December 2021 metagenomes, only three MAGs contained SOX genes. One MAGs only contained the *soxB* gene, one MAG contained the *soxB* gene and the *sqr* gene, and one MAG contained all the SOX genes excluding *soxB*. These three MAGs were also assigned to the HIMB30 genus. Of the three MAGs from the April 2022 metagenomes, two MAGs contained the SOX genes and were assigned to the HIMB30 genus. One MAG contained a complete SOX pathway, whereas the other MAG was missing both *soxC* and *soxD*. The absence of *soxCD* could indicate that this taxon is capable of intracellular sulfur storage [45].

##### Dissimilatory sulfate reduction/ reverse dissimilatory sulfate reduction

Only one MAG found in the 0 m-depth December 2021 sample contained genes from the Dsr/rDSR pathways, specifically *dsrAB* (Fig. 7). This MAG also contained both *sqr* and *fccB* genes and was assigned to the GCF-002020875 order within the Gammaproteobacteria class.

##### Assimilatory sulfate reduction

Only three of the 14 MAGs with sulfur cycling genes contained genes involved in assimilatory sulfate reduction, and these three MAGs were found only in the December 2021 metagenomes (Fig. 7). These MAGs did not contain genes involved in SOX or Dsr/rDSR pathways. One MAG assigned to the Casp-actino5 genus contained *cysNC* and *cysD* genes, as well as *sqr*. Another MAG assigned to the SKUL01 genus contained only the *cysH* gene. The third MAG contained only *cysD* and *cysH* genes and was not given a genus assignment.

#### Phototrophy and carbon fixation in contigs

KO identifiers of genes involved in carbon fixation pathways such as the Calvin-Benson-Bessham cycle (CBB), 3-hydroxypropionate bicycle (3HP), the 3-hydroxypropionate/4-hydroxybutyrate cycle (3HP/4HB), the reductive acetyl-CoA pathway (RAcCoa), the dicarboxylate/4-hydroxybutyrate cycle (DC/4HB), and the reductive tricarboxylic acid cycle (rTCA). Additionally, KO identifiers of genes involved in oxygenic photosynthesis, anoxygenic photosynthesis, and photoheterotrophy were compared within and between each sample metagenome’s contigs.

Genes involved in the CBB, RAcCoa, rTCA, and 3HP cycles were present in the metagenomes (Supplemental Fig. 4). Of these pathways, genes involved in the CBB pathway had the highest relative coverage across all depths and time points. The December 2021 metagenomes appeared to have the highest relative coverage of CBB genes compared to the other time points. However, not all CBB genes were present across depths and time points. For example, sedoheptulose-bisphosphatase was only present in the 10 m-depth metagenome from December 2021 with relatively low coverage. Additionally, Rubisco genes (i.e., ribulose-bisphosphate carboxylase small chain *rbcS* and large chain *rbcL*) phosphoribulokinase (*PRK*), known to be necessary for oxygenic photosynthesis, were found at relatively low coverage across depths and time points.

The rTCA pathway exhibited the highest relative coverage in the August 2021 metagenomes, and the RAcCoa and 3HP pathways exhibited the highest relative coverage across the April 2022 metagenomes. The only pathway that was complete in any metagenome was the 3HP pathway, which appeared to be complete in the 0 m-depth and 5 m-depth metagenomes from August 2021, though the relative coverage of these genes was not equal across the pathway.

Genes involved in Photosystem I and II, anoxygenic photosynthesis, and in bacterial rhodopsin complexes (i.e., sensory rhodopsin, *sop*; beta-carotene 15,15’-dioxygenase associated with proteorhodopsin, *blh*) were found in the metagenomes across depths and time points (Supplemental Fig. 5). Of all the phototrophic genes examined, bacterial rhodopsin genes exhibited the highest relative coverage. The *blh* gene had the highest relative coverage across all depths and time points, with the highest relative coverage in the April 2022 metagenomes. This gene is part of the retinal biosynthesis operon that is required for a functional proteorhodopsin [46]. The *sop* gene also exhibited higher relative coverage than other phototrophic genes (excluding *blh*), with the highest relative coverage across the December 2021 metagenomes. Oxygenic photosynthetic genes, particularly those involved in the Photosystem II (PS II) exhibited higher relative coverage in the December 2021 metagenomes, notably the 0 m-depth and 5 m-depth metagenomes. However, photosystem II P680 reaction center D1 protein (*psbA*), a gene within PS II, exhibited the highest relative coverage in the August 2021 metagenomes, specifically in the 5 m-depth metagenome. Additionally, most of the genes involved in Photosystem I (PS I) were absent across the April 2022 metagenomes. The anoxygenic photosynthesis genes considered here (*pufM*, *pufL*) were both present only in the August 2021 metagenomes, and exhibited lower relative coverage compared to other phototrophic genes.

#### Phototrophy and carbon fixation in MAGs

As was done with the contigs, KO identifiers of genes involved in carbon fixation pathways (i.e., CBB, 3HP, 3HP/4HB, RAcCoa, DC/4HB), and rTCA) and genes involved in various types of phototrophy (i.e., oxygenic photosynthesis, anoxygenic photosynthesis, and photoheterotrophy) were compared within and between the MAGs from each metagenome.

All 19 MAGs contained carbon fixation genes. 17 MAGs contained genes involved in the CBB pathway, yet no MAG contained a complete CBB pathway (Supplemental Fig. 6). Only 1 MAG (i.e., 8.2.21.10 m.bin.22, assigned to the HIMB30 genus) contained the three genes necessary for photosynthesis (i.e., *rbcS*, *rbcL*, *PRK*). This MAG also contained the 9 of the 12 CBB genes examined, the most of any MAG, and contained all the genes in the RAcCoa pathway. In total, 13 of the 19 MAGs contain genes belonging to multiple C fixation pathways. A portion of these MAGs with genes from multiple C fixation pathways were assigned to the HIMB30, Casp-actino5, and M55B157 genera. Overall, the CBB genes exhibited the highest relative coverage of all the C fixation genes considered across the MAGs.

Only one MAG contained genes involved in phototrophy (Supplemental Fig. 7); this MAG (12.22.21.0 m.bin.13) was assigned to the genus HIMB30. This MAG contained *blh* gene at high relative coverage, and the *sop* gene at lower relative coverage. As described above, the *blh* gene contributes to the function of proteorhodopsin and the *sop* gene is involved in the sensory rhodopsin complex. Genes involved in oxygenic and anoxygenic photosynthesis were not found in the MAGs.

## Discussion

The Salton Sea is a hypersaline lake in Southern California that is rapidly shrinking due to evaporation and water diversion. The only water the lake receives is from agricultural runoff, contributing its eutrophic status [1]. The shrinking of the Salton Sea has been associated with an increased frequency in gypsum bloom events [7] as well as an increase in the exposed playa sediment, which is projected to increase local PM concentrations and worsen air quality [9, 13, 14]. The lake’s microbiome is thought to be involved in these ecological interactions, yet previous research only focused on the composition of the surface water [5, 9, 12, 47, 48]. Here, we explore the microbial composition and functional diversity throughout the Salton Sea water column at eight depths across three different seasons. We specifically highlight microbial sulfur cycling genes, notably sulfur oxidation genes, because the Salton Sea’s sulfur dynamics are associated with regional air quality and public health [9, 12]. Collectively our findings suggest that while the relative abundances of halophilic mixotrophs and extremotolerant bacteria in the Salton Sea fluctuate by season, their functional diversity and resiliency allows them to dominate the lake across time and depth.

### Halophiles and halotolerant bacteria are found across time and depth

Hypersaline waterbodies such as the Salton Sea select for halophilic microorganisms that can withstand high osmotic stress [49]. As hypothesized, the major bacterial genera that were present across all time points and depths in the water column included known halophiles such as DS001 of the *Microbacteriaceae* family, *Litoricola* of the *Litoricolaceae* family, *Synechococcus* of the *Synechococcaceae* family, and *Truepera* of the *Trueperaceae* family. These organisms have been found in saline and hypersaline waterbodies around the globe, however, only the cyanobacteria *Synechococcus* had been identified in Salton Seawater samples [50, 51]. Other major bacterial genera we identified, including *Candidatus* Aquiluna, *Brumimicrobium*, CL500-3, *Fluviicola*, and subclade Ia of the SAR11 clade, have been identified in marine and oligohaline conditions, yet have not been associated with hypersaline ecosystems [52–58]. Their presence throughout the water column across seasons, with salinity ranging from 57 – 61.54 ppt in the lake (Table 1; [60]), suggests that these taxa have adapted to the hypersaline conditions of the Salton Sea.

Of the genera we discovered, DS001 was of particular interest because this genus was found with a relative abundance of at least 5% in every sample and had a relative abundance of at least 20% in all samples collected in August 2021 and December 2021. DS001 has been found in hypersaline lakes including the La Brava-La Punta Lake system in the Atacama Desert, Lake Chiprana in the Menegros Desert, and Florida Bay within the Florida Everglades [60–62]. It is also a member of the *Microbacteriaceae* family, which has been found to thrive in hypersaline conditions [49, 63]. Another major genus we found, *Litoricola*, has been identified in the Florida Bay as well as other marine sources like the mariculture ponds in the Shandong province of China, the Xiamen Sea in the Fuijan province of China, and in the East Sea near the Gangwon province of South Korea [61, 64, 65]. This genus is a member of the order *Oceanospirallales*, which contains halophilic and halotolerant taxa [64]. *Truepera*, another major genus, was also isolated from the mariculture ponds in the Shandong province and is known to be facultatively halophilic [65, 66]. Lastly, though some *Synechococcus* are known as freshwater-body cyanobacteria, certain *Synechococcus* strains are known to be euryhaline, indicating that they can withstand a wide range of salinity by producing compatible solutes [67–69]. Collectively these results demonstrate that halotolerance is the most basic requirement for microbial survival within the Salton Sea water column.

### Lake stratification cycle structures microbial communities

Despite the resilience of these halophilic taxa across time and depths, the water column microbiome was affected by seasonal differences in geochemistry. Redundancy analysis and a principal coordinates analysis revealed that not only do microbial communities throughout the water column cluster together by time point, but their compositional differences are driven by temperature, DOM, and %DO. Furthermore, Shannon-Weiner diversity across the water column significantly differed between August 2021 (i.e., as lake stratification subsides) versus December 2021 and April 2022 (i.e., as the water column is mixing and oxygenated), whereas species richness did not exhibit this pattern. This indicated that while the number of bacterial species throughout the water column did not significantly change with the season, the abundance of the present bacterial species did fluctuate. Overall, the microbial community, while resilient across season, responded to the seasonal geochemical transitions associated with fluctuating thermo- and chemoclines. Many dominant microbial genera found in the water column, such as DS001 and *Litoricola*, were observed across seasons and depths, yet their respective relative abundances shifted with their environments over time (Fig. 4). The variability in the relative abundance of the major bacteria we observed hints at the metabolic flexibility required for these taxa to maintain a stronghold in the water column, which we explore later in this manuscript.

### Seasonal lake stratification and sulfur availability select for sulfur oxidation and incomplete sulfate cycling

The fluctuating prevalence of genes in the metagenomes that code for sulfur cycling enzymes paralleled the seasonal variations in the oxycline and sulfur chemoclines that form and dissipate across the water column. Many genes involved in thiosulfate, sulfur, and hydrogen sulfide oxidation pathways were present across the metagenomes and the MAGs, yet the relative coverage of these genes varied by pathway in each season. The functional plasticity and redundancy observed in the Salton Seawater metagenomes and MAGs show that the water column is selecting for functional diversity over taxonomic diversity in the water column microbiome.

Genes coding for proteins involved in hydrogen sulfide oxidation (i.e., oxidize sulfide to polysulfide or sulfur respectively; *sqr, fccB*) and thiosulfate oxidation pathway (i.e., oxidizes thiosulfate [S_2_O_3_^2−^], hydrogen sulfide [H_2_S], elemental sulfur [S_0_], and sulfite [SO_3_^2−^] to sulfate [SO_4_^2−^]; *soxABCDXYZ* or SOX pathway) exhibited the highest relative coverage compared to other sulfur cycling genes in August 2021 (Fig. 6), when the oxycline was still present and H_2_S was at its highest concentration in the hypolimnion, with an average concentration of 23.30 μM. Additionally, two MAGs found in the August 2021 metagenomes contained most of the genes coding for enzymes in the SOX pathway and were assigned to the genus HIMB30, a known halophile capable of sulfide oxidation (Fig. 7; [70, 71]). Our findings suggest that various hydrogen sulfide oxidation strategies are conserved in the microbiome and match the seasonal geochemistry in the water column. Furthermore, we found that sulfide oxidation was most prominent in the 5 m-depth metagenome in August 2021, which corresponds to the depth of the oxic-anoxic interface. The relative coverage of genes assigned to *phsA/psrA*, which performs elemental sulfur/thiosulfate disproportionation (i.e., sulfur compound acts as an electron donor and acceptor), was also found at a relatively higher coverage in the 5 m-depth and 10 m-depth metagenomes in August 2021. This result indicated that microorganisms in the lowest depths of the stratified Salton Sea have the potential for aerotolerance and sulfur disproportionation.

The presence of the *dsrAB* genes in August 2021 metagenomes demonstrates that dissimilatory sulfate reduction (i.e., Dsr) or reverse dissimilatory sulfate reduction (i.e., rDSR) was conserved in the microbiome throughout the water column. It is possible that Dsr was occurring in the lower depths of the lake where the %DO was < 1% (from 9 – 10.5 m, Table 1), or that these genes were used for sulfide oxidation via rDSR. rDSR genes code for Dsr proteins that can work in reverse (i.e., for oxidation, not sulfate reduction) to oxidize sulfite into adenylyl sulfate (i.e., APS) and subsequently SO_4_^2−^ [72–75]. However, *aprAB* genes, which are necessary for Dsr/rDSR [76], were absent from the metagenomes collected at this time (Fig. 6). Moreover, *dsrAB* were observed in August 2021 metagenomes at low relative coverages (Fig. 6). The low coverages of *dsrAB* coupled with the absence of *aprAB* suggests that SO_4_^2−^ reducing and/or sulfur oxidizing bacteria that utilize Dsr/rDSR pathways were not captured by our amplicon and metagenomic sequencing approach. While several of the major bacterial genera observed in August 2021 are members of phyla known to use either/or Dsr/rDSR pathways, their presence across the water column made it difficult to discern if they were able to reduce SO_4_^2−^ in the stratified lake. DS001 and CL500-3 are genera that belong to phyla (Actinobacteriota and Planctomycetota respectively) known to contain the Dsr pathway [77]. Actinobacteriota are capable of Dsr/rDSR, whereas Planctomycetota are capable of sulfate reduction [77]. *Litoricola*, another major genus in August 2021, is a member of the class Gammaproteobacteria*,* which can utilize the rDSR pathway for sulfur oxidation [77, 78]. Assessing the phylogenies of these bacterial genes across these major genera and examining their respective transcription rates would confirm whether Dsr and/or rDSR pathways are actively used by the microbiome as the Salton Sea overcomes stratification [77–79].

As water temperatures cooled by nearly 15 °C in December 2021, SO_4_^2−^ and %DO were at their highest concentrations, and the concentration of H_2_S was greatly reduced. The relative coverages of genes involved in the Dsr/rDSR, notably *aprAB*, were higher than the relative coverages of the SOX genes at this time. Dsr has been occasionally observed in oxygenated conditions [80], however the concentration of SO_4_^2−^ reached its peak in December 2021 (~ 197.57 mM), indicating that sulfate reduction was not probable. Thus, it is unlikely that *dsrAB* and *aprAB* were used for reducing SO_4_^2−^ and instead were used for rDSR. With little H_2_S available to oxidize, these results suggest that intermediates produced via microbial, incomplete sulfate oxidation likely contributed to the high concentrations of SO_4_^2−^ during lake turnover. These results are further supported by the identification of known bacteria that contain the Dsr/rDSR pathways throughout the seasons, but particularly in December 2021. As described above, DS001, the most abundant bacterial genus throughout the water column in December 2021, is a member of the Actinobacteriota phylum, which contains the Dsr pathway and can perform sulfate reduction and/or sulfur oxidation [77]. *Candidatus* Aquiluna, another abundant genus in this season, is also a member of the Actinobacteriota phylum and capable of utilizing Dsr/rDSR genes [77]. *Litoricola* (Gammaproteobacteria*)* and Clade Ia (Alphaproteobacteria*)* are in bacterial classes that can use the rDSR pathway for sulfide oxidation [77, 78]. Collectively, our metagenome and amplicon data reflect that the rDSR pathway is a conserved process in this microbiome. Additionally, *sqr* genes maintained a relatively high coverage in December 2021 across metagenomes, suggesting that microbial polysulfide production continued as the lake mixed. This was also observed in the MAGs found in the December 2021 metagenomes, where 5 of the 9 MAGs with sulfur cycling genes contained *sqr* genes. Previous work has shown that heterotrophic microorganisms containing *sqr* genes and genes that code for persulfide dioxygenase (PDO) can oxidize sulfide in aerobic environments [81]. This further supports that intermediate (i.e., incomplete) SO_4_^2−^ cycling is a useful metabolic strategy during lake mixing, when DO concentrations are high and H_2_S concentrations are low.

By April 2022, SO_4_^2−^ and %DO had decreased from December and chemoclines reemerged, with higher concentrations in the epilimnion and lower concentrations in the hypolimnion. H_2_S concentrations had marginally increased throughout the water column from an average of 2.7 μM in December 2021 to an average of 3.31 μM in April 2022. Relative coverage of genes coding for *fccB* and SOX enzymes had increased and were evenly distributed across all metagenomes. Additionally, two MAGs assigned to the genus HIMB30 found in the April 2022 metagenomes contained almost complete SOX pathways with high relative coverage. Sulfide and thiosulfate oxidation were conserved metabolic pathways in the microbiome in April. Yet, SO_4_^2−^ concentration in the water column had decreased and H_2_S had marginally increased from December to April. Additionally, the water column was still thoroughly oxygenated with a minimum %DO of 51% at the maximum depth of 10.5 m, and genes involved in Dsr/rDSR were absent, suggesting that sulfate reduction or oxidation via Dsr/rDSR was not an important microbial process in April 2022. The decline in SO_4_^2−^ concentrations, as well as the lack of available H_2_S and the absence of the Dsr/rDSR pathways, demonstrates that the water column microbiome had the capacity to utilize incomplete sulfide and thiosulfate oxidation as the lake began to stratify in spring, which is comparable to what we observed in August 2021.

### Functional flexibility and redundancy is required for survival in the Salton Sea

**Fig. 8:**
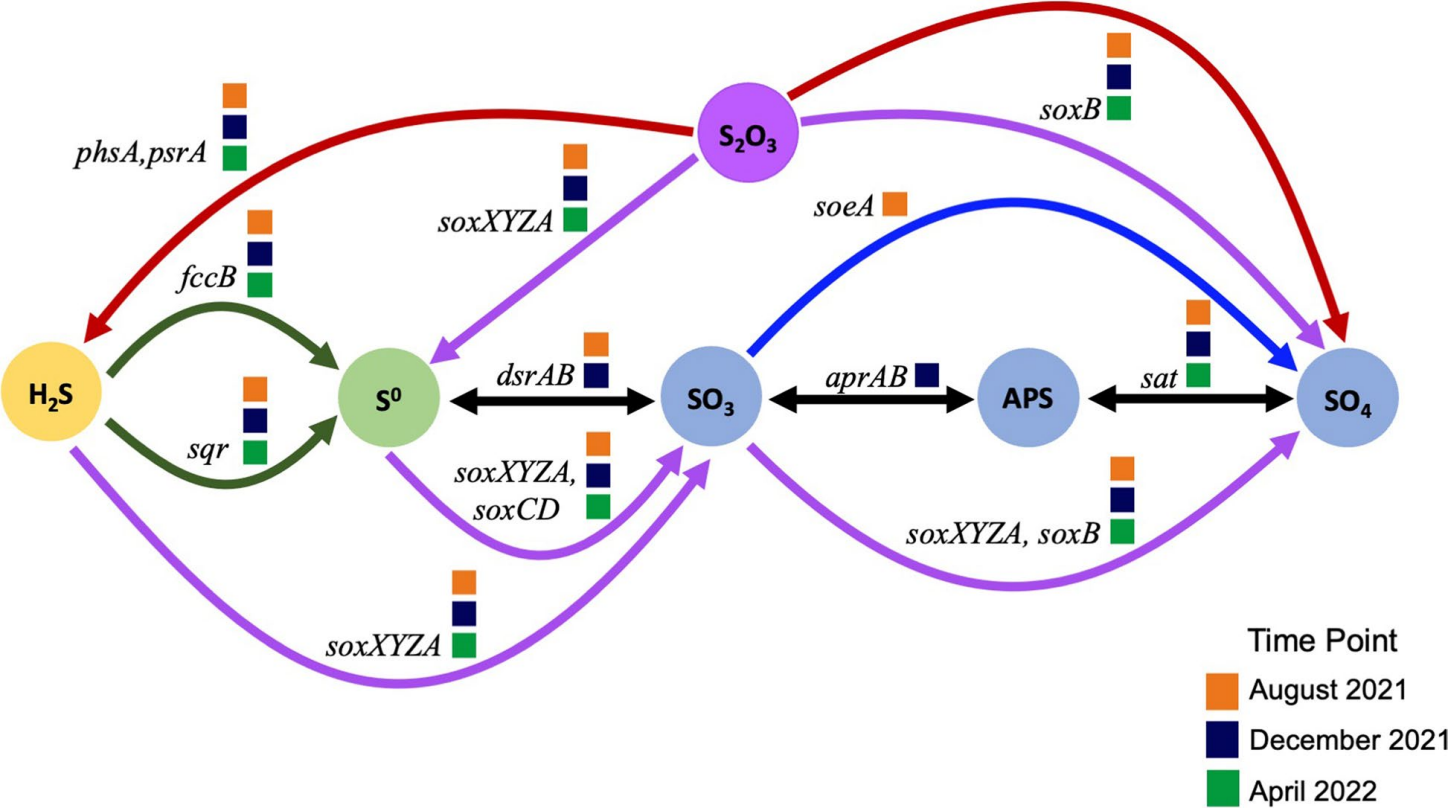
Diagram of Sulfur Oxidation Genes and Sulfur Cycling Pathways Found in the Salton Seawater Metagenomes. This is a diagram that shows the enzymes involved in hydrogen sulfide oxidation (i.e., dark green arrows), sulfur disproportionation (i.e., red arrows), reverse dissimilatory sulfate reduction (i.e., black arrows), thiosulfate oxidation (i.e., purple arrows), and sulfite oxidation (i.e., blue arrow; 49,50,87). The squares next to a gene name indicate if that gene was present in August 2021 (orange), December 2021 (dark blue), and/or April 2022 (green)

Our results suggest that the Salton Seawater microbiome is resilient and well-adapted to this extreme, unstable ecosystem. We see this in the functional redundancy in the sulfur cycling metabolisms in this microbiome and in the set of species that dominate the water column over time (Fig. 8). Eight of our 19 MAGs identified at the genus level were assigned to the genus HIMB30. HIMB30 has been shown to oxidize sulfide, fix CO_2_ via the Calvin-Benson-Bessham (CBB) cycle, and use proteorhodopsin as a means of photoheterotrophy [71]. The functional annotation of the HIMB30 MAGs revealed that they contained genes that code for thiosulfate and hydrogen sulfide oxidation enzymes, enzymes in the CBB cycle, and genes involved in photoheterotrophy (i.e., *blh*, involved in proteorhodopsin function, and *sop* involved in the sensory rhodopsin complex; 65). One MAG assigned to HIMB30 contained the complete SOX pathway (Fig. 7) as well as genes involved in CO_2_ fixation (i.e., *rbcS*, *rbcL*, *PRK;* Supplemental Fig. 6), indicating that this microorganism is chemoautotrophic [83–86]. Savoie et al. found that the clade within Gammaproteobacteria that houses HIMB30, OM252, is capable of alternating between chemoorganoheterotrophic or chemolithoautotrophic growth depending on their environment. Furthermore, HIMB30 is closely related to *Litoricola*, which we identified as a major genus in the Salton Sea [70]. Recent research has revealed that *Litoricola*, which was previously thought to be a chemoheterotroph [64], contains sulfur oxidation genes (i.e., *soxAX* genes), genes involved in oxygenic photosynthesis (i.e., RuBisCO, carbon monoxide dehydrogenase; *rbcL*, *coxL*), and genes involved in photoheterotrophy (i.e., rhodopsin genes) in their genomes [84, 86]. The presence of HIMB30 MAGs, their sulfur and carbon cycling genes, and the overwhelming presence of *Litoricola* across seasons highlights the metabolic flexibility in this sulfate-rich system as a means of survival and ecological resilience. Other dominant genera we observed across seasons, *Synechococcus* and *Trupera*, are known to adapt to their surroundings. *Synechococcus* is a globally distributed cyanobacteria that can survive in low light conditions if the bacteria can absorb far-red light via a process known as low-light photoacclimation [87]. *Truepera* is facultatively halophilic thermophile that has been found in hypersaline lakes, marine environments, and hot springs [66, 88, 89]. This genus is resistant to ionizing radiation and osmotic stress, which may contribute to its ability to persist despite extreme changes in salinity [66, 90]. Considering that these taxa were found throughout lake stratification and mixing, it is reasonable to surmise that the halophilic microorganisms in the Salton Sea have the functional flexibility and redundancy necessary to survive the intense selection pressures of this extreme, variable ecosystem.

Typically, functional convergence is observed with taxonomic variation within environmental microbiomes, particularly across redox gradients and depth [91–93]. Geochemical gradients such as changes in salinity, oxygen availability, and carbon availability serve as selective pressures for specific bacteria that have the functional capacity to endure these conditions; thus, functional diversity is a more useful indicator of microbial survival compared to phylogenetic diversity [92, 93]. Taxonomic variation and functional redundancy or niche partitioning along a gradient have been observed in other saline and hypersaline systems beyond the Salton Sea [94–96]. However, the Salton Sea offers a unique insight into the selection of microbial trophic pathways because it is a terminal, hypersaline lake that receives a consistent influx of agricultural runoff, and it is rapidly shrinking due to water diversion [4, 13]. This, along with strong, seasonal winds in the region [9], select for extremophilic and extremotolerant traits in the volatile, shallow Salton Sea. While shallow water bodies are less susceptible to lake stratification [97], the Salton Sea (with a depth of 10.5 m at the time of this study) can be stratified for months each year until temperatures in the region finally cool enough for lake turnover to occur [6, 7]. The shallow depths coupled with prolonged stratification could limit the spatial dispersal of the microorganisms in the water column. This environmental filtering may explain why we observed a set of dominant bacteria whose abundance consistently fluctuated over time, contributing to significant differences in seasonal, microbial composition. Moreover, we observed both functional diversity and redundancy of sulfur cycling genes within the metagenomes, underscoring the importance of trait selection over phylogeny in microbiome assembly [92, 93, 98]. The microbial functional diversity of the Salton Sea speaks to the strong evolutionary pressures that confer survival in this degrading ecosystem.

### The lake water and dust connection

Mineral signatures from the lake water such as SO_4_^2−^ have been identified in playa dust collected in the region. Frie et al. (2019) found that CaSO_4_ and MgSO_4_ consistently dominated dust from the Salton Sea playa, suggesting that these minerals are indicative of wind erosion and dust production at the playa surface [9]. As we previously described, the occurrence of gypsum blooms in the Salton Sea has been connected to increased hospitalizations in the area, particularly hospitalizations associated with respiratory distress [12]. These findings connect the Salton Sea’s sulfur cycle to the emissivity and composition of the dust in this region. Thus, given that the Salton Seawater microbiome is involved in the ecosystem’s sulfur cycle, it is plausible that these microorganisms also play a role in structuring the chemical and microbial composition of the Salton Sea dust. Microorganisms can traverse the atmosphere as free cells or attached to particulates and can become entrained in the dust as sea spray and playa sediment become airborne [99]. In addition, microorganisms originating from marine sources have been found in dust and cloud water, indicating that certain microorganisms can transition from water bodies to dust and beyond [100, 101]. The metabolic flexibility and environmental tolerance exhibited by the Salton Seawater microbiome may allow these microorganisms to withstand the oxidative and osmotic stresses of the atmosphere upon entry from the lake’s sea spray or playa [102–104]. While the interface between the lake water and the atmosphere has been explored, more research is required to investigate the microbial composition of the Salton Sea dust and its relationship to the seasonal dynamics of the lake and its microbiome. Moreover, while it has been established that dust from the Salton Sea induces pulmonary inflammation, the microbial composition of Salton Sea dust aerosols has not been characterized [10, 11]. Understanding the microbial interactions between the lake and the dust is increasingly urgent due to the rapid shrinking of the Salton Sea, which is expected to alter the seasonal stratification and sulfur cycle in this ecosystem, and thus, change the biogeochemistry of the playa sediment that enters the atmosphere [3, 13]. Additionally, understanding the drivers of the Salton Sea microbiome, and how these microorganisms and their metabolites become airborne and disperse, can inform restoration and remediation strategies aimed at reducing harmful dust emissions in the region. Thorough investigation into the microbial interactions of the Salton Sea region across its substrates is required to holistically address and mitigate the unfolding public health crisis at the Salton Sea [12, 105–107].

## Conclusion

In this study, we have elucidated the assembly and functional diversity of the microbiome within the Salton Sea water column, which has not been previously explored. Despite the significant seasonal changes in microbiome composition, halophilic mixotrophs (i.e., organisms that can alternate between chemorganotrophy and chemolithoautrophy) and other metabolically flexible bacteria continued to dominate the microbial water column community. Metagenomes collected throughout the water column revealed that a variety of sulfur oxidation strategies were shared by the microorganisms in the lake, notably thiosulfate oxidation via the SOX pathway, sulfide oxidation via *sqr* and *fccB*, the reverse dissimilatory sulfate reduction pathway, and the sulfur disproportionation pathway. The prominence of the thiosulfate and hydrogen sulfide oxidation genes specifically was also reflected in the MAGs, with many of these MAGs being assigned to a known bacterial mixotroph capable of sulfur oxidation, HIMB30. Our results highlight the functional versatility and redundancy in sulfur cycling pathways, namely sulfur oxidation, that was conserved in the Salton Seawater microbiome over time. Further work is needed to determine exactly when and how these microorganisms alternate between sulfur oxidation and reduction pathways throughout the Salton Sea water column, ideally using metatranscriptomics, to clarify how the microbiome shifts its trophic strategies to live in this dynamic, shrinking lake. Furthermore, understanding the expression of these sulfur-based metabolisms in the Salton Seawater microbiome may shed light on the pathological impact of gypsum blooms and playa dust on the local population [12]. Overall, our findings show that the water column microbiome within the Salton Sea is intimately involved with its seasonal nutrient cycling and redox structure and thus this ecosystem’s function and stability.

## Supplementary Information

Supplementary Material 1.

## Data Availability

Sequence data that support the findings of this research have been deposited in the Sequence Read Archive with the primary accession code PRJNA1153586. Bash and R scripts used to process and analyze the sequence data for this project can be found in this GitHub repository: https://github.com/hlfreund/SaltonSeaWaterMicrobiome.

## Abbreviations

SO_4_^2^^−^: Sulfate
H_2_S: Hydrogen sulfide
%DO: Percent saturation of dissolved oxygen
ORP: Oxidative-reduction potential
DOM: Dissolved organic matter
MAG: Metagenome-assembled genome
SOX: Thiosulfate oxidation
Dsr: Dissimilatory sulfate reduction
rDSR: Reverse dissimilatory sulfate reduction
