# Supplementary material for: Diversity of sulfur cycling halophiles within the Salton Sea, California’s largest lake
Source: BMC Microbiol. 2025 Mar 6;25:120. doi: 10.1186/s12866-025-03839-2 (PMC11883979; doi:10.1186/s12866-025-03839-2)
Supplement: Supplementary file 1 — Supplementary Material 1. [file 12866_2025_3839_MOESM1_ESM.docx]

Supplemental Information

**Title**: Diversity of Sulfur Cycling Halophiles within the Salton Sea, California’s Largest Lake

**Authors**: Freund, L.^1^, Hung, Caroline^2^, Topacio, Talyssa M.^3^, Diamond, Charlie^2^, Fresquez, Alyson^2^, Lyons, Timothy W.^2^, and Aronson, Emma L.^3^

1. Genetics, Genomics, and Bioinformatics Program, University of California, Riverside, CA
2. Department of Earth and Planetary Sciences, University of California, Riverside, CA
3. Department of Microbiology and Plant Pathology, University of California, Riverside, CA

**Supplemental Figures**

**

**

**Figure S1. Total Amplicon Sequence Variants (ASVs) by Sample.** This bar plot shows the total number of unfiltered ASVs per sample by Sample ID, organized by their respective totals.

**Figure S2. Vertical Profiles of (A) Shannon-Weiner Diversity and (B) Species Richness**. These scatter plots show the Shannon-Weiner diversity and species richness in each sample by time point and depth. Each point represents a sample and are colorized by the time point they were collected: August 2021 (orange), December 2021 (blue), and April 2022 (green).

**Figure S3. Bacterial Genera by Sample Depth and Collection Date**. These stacked bar plots display the relative abundance of bacterial genera (16S rRNA) with a relative abundance of less than 1%. These plots are separated by their sampling dates: August 2021 (**A**), December 2021 (**B**), and April 2022 (**C**). The x-axis contains the samples organized in order by date and increasing depth from left to right, and the y-axis is the relative abundance.

**

**

**Figure S4. Carbon Cycling in the Metagenomes by Depth and Collection Date.** This heat map shows the relative coverage (i.e., center-log ratio transformed coverage) of genes assigned to KOs involved in carbon metabolism. Each column is a metagenome, organized from left to right by collection date and depth (0m, 5m, and 10m). The carbon cycling genes are broken up by their pathways: 3-Hydroxypropionate Bi-cycle (i.e., 3HP), Reductive Tricarboxylic Acid Cycle (i.e., rTCA), Reductive acetyl-CoA Pathway (i.e., RAcCoa), and the Calvin-Benson-Bassham cycle (i.e., CBB). Gray squares indicate that the gene was absent.

**

**

**Figure S5. Phototrophy in the Metagenomes by Depth and Collection Date.** This heat map shows the relative coverage (i.e., center-log ratio transformed coverage) of genes assigned to KOs involved in various types of phototrophy. Each column is a metagenome, organized from left to right by collection date and depth (0m, 5m, and 10m). The phototrophy genes are broken up by their phototrophic systems/pigments: proteorhodopsin (i.e., PR), sensory rhodopsin (i.e., SR), oxygenic photosynthesis Photosystem II (i.e., PS II), oxygenic photosynthesis Photosystem I (i.e., PS I), and anoxygenic photosynthesis (i.e., AnOx PS). Gray squares indicate that the gene was absent.





**Figure S6. Carbon Cycling in the Metagenome-Assembled Genomes (MAGs) by Depth and Collection Date.** This heat map shows the relative coverage (i.e., center-log ratio transformed coverage) of genes assigned to KOs involved in carbon metabolism in the MAGs found. Each column is a MAG, organized from left to right by collection date and depth (0m, 5m, and 10m). The carbon cycling genes are broken up by their pathways: Gray squares indicate that the gene was absent.





**Figure S7. Phototrophy in the Metagenome-Assembled Genomes (MAGs) by Depth and Collection Date.** This heat map shows the relative coverage (i.e., center-log ratio transformed coverage) of genes assigned to KOs involved in various types of phototrophy. Each column is a MAG, organized from left to right by collection date and depth (0m, 5m, and 10m). The phototrophy genes are broken up by their phototrophic systems/pigments: proteorhodopsin (i.e., PR), sensory rhodopsin (i.e., SR), oxygenic photosynthesis Photosystem II (i.e., PS II), oxygenic photosynthesis Photosystem I (i.e., PS I), and anoxygenic photosynthesis (i.e., AnOx PS). Gray squares indicate that the gene was absent.

| **SampleID** | **Bin**  **Num** | **Taxa**  **Level** | **Marker**  **Lineage** | **Lineage**  **ID** | **Genome**  **Num** | **Completeness** | **Contamination** | **Strain**  **Heterogeneity** | **GC**  **Content** |
| --- | --- | --- | --- | --- | --- | --- | --- | --- | --- |
| 8.24.21.0m | bin.15 | c | Gammaproteobacteria | (UID4443) | 356 | 94.51 | 0.74 | 33.33 | 53.4 |
| 8.24.21.5m | bin.21 | k | Bacteria | (UID1453) | 901 | 92.31 | 4.7 | 88.89 | 64.2 |
| 8.24.21.10m | bin.22 | c | Gammaproteobacteria | (UID4443) | 356 | 94.51 | 0.56 | 33.33 | 53.3 |
| 12.22.21.0m | bin.11 | k | Bacteria | (UID1453) | 901 | 92.31 | 1.92 | 66.67 | 66.7 |
| 12.22.21.0m | bin.13 | c | Gammaproteobacteria | (UID4443) | 356 | 94.14 | 1.11 | 25 | 53.3 |
| 12.22.21.0m | bin.7 | c | Gammaproteobacteria | (UID4274) | 112 | 91.32 | 0.46 | 100 | 57 |
| 12.22.21.5m | bin.3 | k | Bacteria | (UID1453) | 901 | 93.16 | 0.85 | 0 | 66.7 |
| 12.22.21.5m | bin.30 | c | Gammaproteobacteria | (UID4443) | 356 | 94.51 | 0.37 | 0 | 53.3 |
| 12.22.21.10m | bin.17 | c | Gammaproteobacteria | (UID4443) | 356 | 94.14 | 0.37 | 0 | 53.4 |
| 12.22.21.10m | bin.2 | k | Bacteria | (UID2569) | 434 | 97.04 | 0.81 | 100 | 46 |
| 12.22.21.10m | bin.6 | k | Bacteria | (UID2569) | 434 | 96.92 | 4.4 | 0 | 39.5 |
| 12.22.21.10m | bin.7 | c | Gammaproteobacteria | (UID4274) | 112 | 91.4 | 0.09 | 100 | 57 |
| 4.13.22.0m | bin.16 | c | Gammaproteobacteria | (UID4443) | 356 | 93.28 | 0.74 | 33.33 | 53.3 |
| 4.13.22.0m | bin.22 | c | Gammaproteobacteria | (UID4201) | 1164 | 97.13 | 1.72 | 66.67 | 43.2 |
| 4.13.22.5m | bin.19 | c | Gammaproteobacteria | (UID4201) | 1164 | 92.43 | 1.05 | 66.67 | 43.3 |
| 4.13.22.5m | bin.24 | c | Gammaproteobacteria | (UID4443) | 356 | 94.69 | 0.37 | 0 | 53.3 |
| 4.13.22.5m | bin.5 | o | Actinomycetales | (UID1663) | 488 | 93.99 | 2.88 | 25 | 64.7 |
| 4.13.22.10m | bin.18 | c | Gammaproteobacteria | (UID4201) | 1164 | 94.01 | 2.3 | 25 | 43.3 |
| 4.13.22.10m | bin.8 | c | Gammaproteobacteria | (UID4443) | 356 | 95.62 | 0.37 | 0 | 53.3 |

**Table S1. CheckM Results for Metagenome-Assembled Genomes (MAGs) Bin Assignments**. This table contains the CheckM results for each putative, high-quality MAG bin assignment.

| **Sample ID** | **Shannon Wiener Entropy** | **Shannon Weiner Diversity** | **Species Richness** |
| --- | --- | --- | --- |
| 8.24.21.0m | 4.30307865 | 73.9270391 | 1194 |
| 8.24.21.10.5m | 4.55391783 | 95.0038891 | 1369 |
| 8.24.21.10m | 4.51325818 | 91.2185412 | 1276 |
| 8.24.21.3m | 4.21097987 | 67.4225727 | 1228 |
| 8.24.21.4m | 4.26386379 | 71.0841076 | 1242 |
| 8.24.21.5m | 4.41860564 | 82.9804998 | 1270 |
| 8.24.21.7m | 4.20000386 | 66.6865887 | 1021 |
| 8.24.21.9m | 3.7497824 | 42.5118302 | 507 |
| 12.22.21.0m | 4.58879386 | 98.3757033 | 872 |
| 12.22.21.10.5m | 4.65415079 | 105.019998 | 996 |
| 12.22.21.10m | 4.57793663 | 97.313393 | 1070 |
| 12.22.21.3m | 4.70919327 | 110.962607 | 954 |
| 12.22.21.4m | 4.61605442 | 101.094368 | 1040 |
| 12.22.21.5m | 4.63812073 | 103.349942 | 929 |
| 12.22.21.7m | 4.70742802 | 110.766903 | 990 |
| 12.22.21.9m | 4.12722217 | 62.0054428 | 796 |
| 4.13.22.0m | 4.53543507 | 93.2640825 | 847 |
| 4.13.22.10.5m | 4.83553778 | 125.906275 | 861 |
| 4.13.22.10m | 4.64441814 | 104.002833 | 940 |
| 4.13.22.3m | 4.29109902 | 73.046704 | 740 |
| 4.13.22.4m | 4.48281071 | 88.4830236 | 829 |
| 4.13.22.5m | 4.72598945 | 112.842095 | 788 |
| 4.13.22.7m | 4.74851125 | 115.412336 | 906 |
| 4.13.22.9m | 4.2845478 | 72.5697233 | 749 |

**Table. S2. Bacterial Alpha Diversity and Species Richness by Sample.** This table shows the Shannon-Weiner entropy, Shannon-Weiner diversity, and species richness for each sample.

|  | **Comparisons by Time Point** | **P_adj_ value** |
| --- | --- | --- |
| **Shannon-Weiner Diversity** | December 2021 vs August 2021 | 0.027 |
|  | April 2022 vs August 2021 | 0.018 |
|  | April 2022 vs December 2021 | 0.983 |
| **Species Richness** | December 2021 vs August 2021 | 0.312 |
|  | April 2022 vs August 2021 | 0.442 |
|  | April 2022 vs December 2021 | 1 |

**Table. S3. Pairwise Comparison of the Variance in**

**Alpha Diversity and Species Richness.** A post hoc Tukey test was

used to compare the variance in Shannon-Weiner diversity between

collection dates, and a Dunn test was used to compare the

variance in species richness between collection dates.

| **Comparisons by Time Point** | **DF** | **Sums of Squares** | **F Model** | **R^2^** | **P value** | **P_adj_ value** |
| --- | --- | --- | --- | --- | --- | --- |
| December 2021 vs April 2022 | 1 | 7006.287 | 17.13323 | 0.5503196 | 0.001 | 0.003 |
| December 2021 vs August 2021 | 1 | 7416.531 | 13.48028 | 0.4905437 | 0.001 | 0.003 |
| April 2022 vs August 2021 | 1 | 7987.429 | 15.15788 | 0.5198554 | 0.002 | 0.006 |

**Table. S4. Pairwise PERMANOVA Results Comparing Beta Diversity by Collection Date.** This is a pairwise permutational multivariate analysis of variance (PERMANOVA) comparing the variance in beta diversity between collection dates.

| **Site(s)** | **Model** | **Variance** | **F value** | **P value** | **P_adj_ value** |
| --- | --- | --- | --- | --- | --- |
| All | Temperature °C + DOM + %DO | 335.51 | 13.8841 | 0.001 | 0.004 |
|  |  | 169.84 | 7.0285 | 0.001 | 0.004 |
|  |  | 113.20 | 4.6845 | 0.001 | 0.004 |
| August 2021 | DOM | 157.93 | 1.8571 | 0.001 | 0.002 |
| December 2021 | ORP | 77.39 | 1.3089 | 0.002 | 0.012 |
| April 2022 | DOM | 60.08 | 1.107 | 0.026 | 0.068 |

**Table. S5. Redundancy Analysis Results of Microbial Composition Across and Within Collection Dates.** These results show which environmental variables were significant drivers of beta diversity across all three collection dates and within each collection date based on a redundancy analysis (RDA).

**Table S6. Taxonomic Annotation of Metagenome Assembled Genomes (MAGs).** This table contains the taxonomic annotation results from GTDB-tk of high-quality bins identified in this project.

| **Sample ID** | **Domain** | **Phylum** | **Class** | **Order** | **Family** | **Genus** | **Species** |
| --- | --- | --- | --- | --- | --- | --- | --- |
| 8.24.21.0m.bin.15 | Bacteria | Proteobacteria | Gammaproteobacteria | Pseudomonadales | Litoricolaceae | HIMB30 | unknown |
| 8.24.21.5m.bin.21 | Bacteria | Actinobacteriota | Acidimicrobiia | Acidimicrobiales | Ilumatobacteraceae | Casp-actino5 | unknown |
| 8.24.21.10m.bin.22 | Bacteria | Proteobacteria | Gammaproteobacteria | Pseudomonadales | Litoricolaceae | HIMB30 | unknown |
| 12.22.21.0m.bin.7 | Bacteria | Proteobacteria | Gammaproteobacteria | GCF-002020875 | GCF-002020875 | Unknown | unknown |
| 12.22.21.0m.bin.11 | Bacteria | Actinobacteriota | Acidimicrobiia | Acidimicrobiales | Ilumatobacteraceae | Casp-actino5 | Casp-actino5 sp017859785 |
| 12.22.21.0m.bin.13 | Bacteria | Proteobacteria | Gammaproteobacteria | Pseudomonadales | Litoricolaceae | HIMB30 | unknown |
| 12.22.21.5m.bin.3 | Bacteria | Actinobacteriota | Acidimicrobiia | Acidimicrobiales | Ilumatobacteraceae | Casp-actino5 | Casp-actino5 sp017859785 |
| 12.22.21.5m.bin.30 | Bacteria | Proteobacteria | Gammaproteobacteria | Pseudomonadales | Litoricolaceae | HIMB30 | unknown |
| 12.22.21.10m.bin.2 | Bacteria | Bacteroidota | Bacteroidia | Flavobacteriales | Cryomorphaceae | SKUL01 | unknown |
| 12.22.21.10m.bin.6 | Bacteria | Bacteroidota | Bacteroidia | Flavobacteriales | Crocinitomicaceae | Unknown | unknown |
| 12.22.21.10m.bin.7 | Bacteria | Proteobacteria | Gammaproteobacteria | GCF-002020875 | GCF-002020875 | Unknown | unknown |
| 12.22.21.10m.bin.17 | Bacteria | Proteobacteria | Gammaproteobacteria | Pseudomonadales | Litoricolaceae | HIMB30 | unknown |
| 4.13.22.0m.bin.16 | Bacteria | Proteobacteria | Gammaproteobacteria | Pseudomonadales | Litoricolaceae | HIMB30 | unknown |
| 4.13.22.0m.bin.22 | Bacteria | Proteobacteria | Gammaproteobacteria | CACEW01 | Unknown | Unknown | unknown |
| 4.13.22.5m.bin.5 | Bacteria | Actinobacteriota | Actinomycetia | Nanopelagicales | S36-B12 | M55B157 | unknown |
| 4.13.22.5m.bin.19 | Bacteria | Proteobacteria | Gammaproteobacteria | CACEW01 | Unknown | Unknown | unknown |
| 4.13.22.5m.bin.24 | Bacteria | Proteobacteria | Gammaproteobacteria | Pseudomonadales | Litoricolaceae | HIMB30 | unknown |
| 4.13.22.10m.bin.8 | Bacteria | Proteobacteria | Gammaproteobacteria | Pseudomonadales | Litoricolaceae | HIMB30 | unknown |
| 4.13.22.10m.bin.18 | Bacteria | Proteobacteria | Gammaproteobacteria | CACEW01 | Unknown | Unknown | unknown |
| 8.24.21.0m.bin.15 | Bacteria | Proteobacteria | Gammaproteobacteria | Pseudomonadales | Litoricolaceae | HIMB30 | unknown |
| 8.24.21.5m.bin.21 | Bacteria | Actinobacteriota | Acidimicrobiia | Acidimicrobiales | Ilumatobacteraceae | Casp-actino5 | unknown |
| 8.24.21.10m.bin.22 | Bacteria | Proteobacteria | Gammaproteobacteria | Pseudomonadales | Litoricolaceae | HIMB30 | unknown |
| 12.22.21.0m.bin.7 | Bacteria | Proteobacteria | Gammaproteobacteria | GCF-002020875 | GCF-002020875 | Unknown | unknown |
| 12.22.21.0m.bin.11 | Bacteria | Actinobacteriota | Acidimicrobiia | Acidimicrobiales | Ilumatobacteraceae | Casp-actino5 | Casp-actino5 sp017859785 |
| 12.22.21.0m.bin.13 | Bacteria | Proteobacteria | Gammaproteobacteria | Pseudomonadales | Litoricolaceae | HIMB30 | unknown |
| 12.22.21.5m.bin.3 | Bacteria | Actinobacteriota | Acidimicrobiia | Acidimicrobiales | Ilumatobacteraceae | Casp-actino5 | Casp-actino5 sp017859785 |
| 12.22.21.5m.bin.30 | Bacteria | Proteobacteria | Gammaproteobacteria | Pseudomonadales | Litoricolaceae | HIMB30 | unknown |
| 12.22.21.10m.bin.2 | Bacteria | Bacteroidota | Bacteroidia | Flavobacteriales | Cryomorphaceae | SKUL01 | unknown |
| 12.22.21.10m.bin.6 | Bacteria | Bacteroidota | Bacteroidia | Flavobacteriales | Crocinitomicaceae | Unknown | unknown |
| 12.22.21.10m.bin.7 | Bacteria | Proteobacteria | Gammaproteobacteria | GCF-002020875 | GCF-002020875 | Unknown | unknown |
| 12.22.21.10m.bin.17 | Bacteria | Proteobacteria | Gammaproteobacteria | Pseudomonadales | Litoricolaceae | HIMB30 | unknown |
| 4.13.22.0m.bin.16 | Bacteria | Proteobacteria | Gammaproteobacteria | Pseudomonadales | Litoricolaceae | HIMB30 | unknown |
| 4.13.22.0m.bin.22 | Bacteria | Proteobacteria | Gammaproteobacteria | CACEW01 | Unknown | Unknown | unknown |
| 4.13.22.5m.bin.5 | Bacteria | Actinobacteriota | Actinomycetia | Nanopelagicales | S36-B12 | M55B157 | unknown |
| 4.13.22.5m.bin.19 | Bacteria | Proteobacteria | Gammaproteobacteria | CACEW01 | Unknown | Unknown | unknown |
